# Severity influences categorical likelihood communications: A case study with Southeast Asian weather forecasters

**DOI:** 10.1038/s41598-024-64399-5

**Published:** 2024-06-25

**Authors:** Alice Liefgreen, Sarah C. Jenkins, Sazali Osman, Lorenzo A. Moron, Maria Cecilia A. Monteverde, Esperanza O. Cayanan, Lam Hoang, Diep Quang Tran, Huong Ngo, Agie Wandala Putra, Riefda Novikarany, Sefri Ayuliana, Rebecca Beckett, Adam J. L. Harris

**Affiliations:** 1 Department of Experimental Psychology, University College London, 26 Bedford Way, London, WCH1 0AP UK; 2 Department of Language and Cognition, University College London, 2 Wakefield Street, London, WC1N 1PF UK; 3 Centre for Decision Research, Leeds University Business School, Maurice Keyworth Building, University of Leeds, Leeds, LS2 9JT UK; 4 Met Office, FitzRoy Road, Exeter, Devon EX1 3PB UK; 5 Department of Irrigation and Drainage Malaysia, National Flood Forecasting and Warning Centre, 50480 Kuala Lumpur, Malaysia; 6 Department of Science and Technology, Philippine Atmospheric, Geophysical and Astronomical Services Administration (PAGASA), PAGASA Science Garden Complex, BIR Road, Brgy. Central, 1100 Quezon City, Metro Manila Philippines; 7 Vietnam National Center for Hydro-Meteorological Forecasting, 8 Phao Dai Lang Street, Lang Thuong Ward, Dong Da District, Ha Noi City, Vietnam; 8 The Agency for Meteorology, Climatology, and Geophysics of the Republic of Indonesia (Badan Meteorologi, Klimatologi, dan Geofisika (BMKG), Jl. Angkasa, 2 Kemayoran Jararta Pusat, DKI, Jakarta, 10610 Indonesia

**Keywords:** Impact-based warnings, Risk perception, Risk communication, Severity effect, Natural hazards, Asymmetric loss functions, Environmental impact, Psychology, Natural hazards

## Abstract

Risk assessments are common in multiple domains, from finance to medicine. They require evaluating an event’s potential severity and likelihood. We investigate the possible dependence of likelihood and severity within the domain of impact-based weather forecasting (IBF), following predictions derived from considering asymmetric loss functions. In a collaboration between UK psychologists and partners from four meteorological organisations in Southeast Asia, we conducted two studies (*N* = 363) eliciting weather warnings from forecasters. Forecasters provided warnings denoting higher likelihoods for high severity impacts than low severity impacts, despite these impacts being described as having the same explicit numerical likelihood of occurrence. This ‘Severity effect’ is pervasive, and we find it can have a continued influence even for an updated forecast. It is additionally observed when translating warnings made on a risk matrix to numerical probabilities.

## Introduction

Effective decision making requires a consideration of the utility of an action or inaction’s consequences, and the likelihood of those consequences^1,2^. Risk analysts identify potential consequences and assess their likelihood, in order to guide subsequent decisions: financial risk analysts consider costs associated with different investment decisions, with more weight given to those that are more likely; medical consultants provide a risk analysis service to patients in outlining the potential costs and benefits of a treatment. Investors and patients then utilise this information to decide on investment strategies and treatment options. Impact-based weather forecasting (IBF)^3,4^ sees weather forecasters acting as risk analysts, communicating the severity and likelihood of impacts associated with a weather event (e.g. heavy rainfall) (impact-based warnings [IBW]). In this paper, we test whether the severity of impacts influences the likelihood communicated in weather forecasters’ warnings.

A non-independence between severity and likelihood has previously been observed for interpretations of likelihood information. Resembling a ‘Severity effect’, verbal probability expressions (VPEs) referring to more severe outcomes are perceived as more likely to occur than those referring to neutral outcomes. For instance, when an “unlikely, perhaps very unlikely” sea level rise would cause an island to disappear, higher numerical translations were observed for the expression (“unlikely…”) versus when this island was protected by high cliffs^5^. This effect was also demonstrated in health and forensic contexts^6–9^. This Severity effect is not limited to interpretations of VPEs, but also extends to interpretations of numerical probability expressions across various formats (e.g. percentages and ratios^10^), as well as estimates of visually presented probabilities^11^. One possible explanation for these findings is asymmetric loss function sensitivity^12,13^: when estimating probabilities, individuals are sensitive to different costs associated with different estimation errors. The greater the disutility of an event, the costlier the errors of underestimating its probability often are. In such situations, overestimates provide protection against costly underestimates^13–15^. Asymmetric loss functions are expected to have a particular influence on *communications* of likelihood estimates^5^. Since others might not recognise the severity of a given situation, overestimating likelihoods can increase the chance of appropriate action (as judged by the communicator) being taken.

A number of organisations advise, or require, that probabilistic information in risk communications is provided in the form of verbal categories (e.g. Refs.^16,17^). Consequently, a relevant applied question is whether a Severity effect is observed when translating numbers into verbal categories. If communicators are sensitive to asymmetric loss functions, we should expect to observe a Severity effect. Alternatively, communicators might recognise the potential for expressions to be interpreted as denoting higher probabilities for severe events and hence use lower likelihood categories when referring to severe events. Note that, whilst the latter result has been previously observed, this is within one-to-one communications where politeness concerns (utilising a likelihood expression to soften the impact of bad news) are salient^5,18,19^. In large-scale, ‘official’, risk communications we expect that such concerns are less relevant (see also Ref.^5^).

The complexity of effective risk communication is often exacerbated by the dynamic nature of the risk environment. New and (at times) contradictory information often needs to be integrated into an overall risk assessment, leading to multiple re-assessments over the course of an event (Ref.^20^; e.g. when tracking a storm with changing force and trajectory). This is an inherent aspect of communicating about natural hazards and extreme weather events, which, between 2000 and 2019, claimed approximately 1.23 million lives, affected over 4 billion people, and resulted in economic losses of around US$ 2.97 trillion worldwide^21^. In this paper, across two studies (Pilot and subsequent Main study) we focus on the Southeast Asia region, recruiting weather professionals (scientists and forecasters) from Indonesia and the Philippines (both studies), Malaysia and Vietnam (main study). Southeast Asia has one of the world’s highest exposure rates to natural hazards and extreme weather events, resulting in huge losses of life and extensive damage^22^. One step towards reducing the adverse consequences of such events has been the World Meteorological Organisation’s (WMO) implementation of IBF^3,4^. IBF advocates a shift in focus from what the weather will be, to what the weather will do, with warnings based on the forecasted likelihood and severity of the impacts associated with upcoming meteorological hazards. This approach is intended to facilitate preparatory actions and ultimately reduce the impact of such hazards^23^.

**Figure 1:**
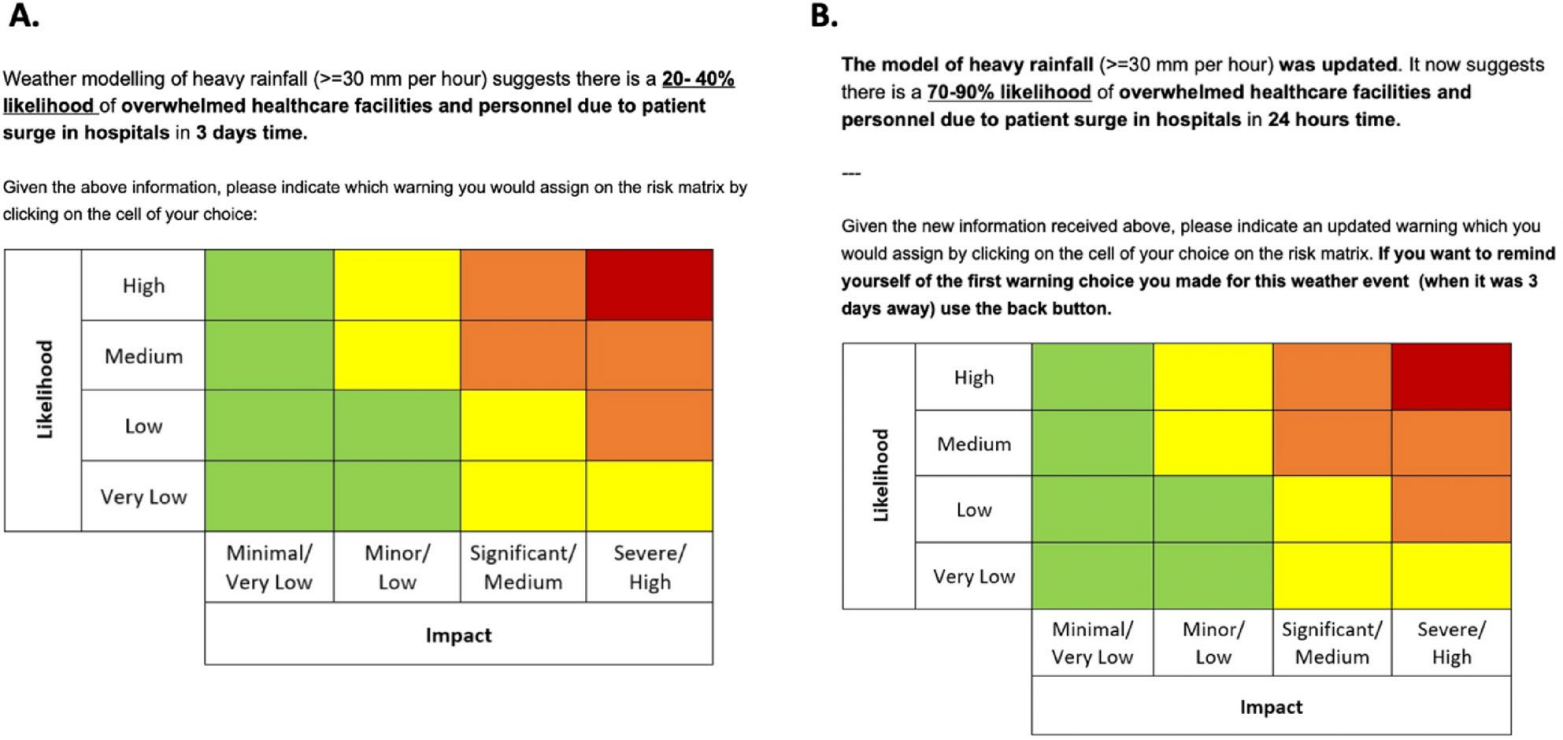
(**A**) Trial in Main study depicting a sequential forecasting scenario with an event of severe impact and low likelihood 3 days away (**B**) developing into severe impact and high likelihood 24 h from the event. Participants indicated a weather warning by clicking on any cell in the presented WMO’s risk matrix.

In order to communicate risks associated with weather hazards, the WMO developed a risk matrix for use in IBWs to communicate the forecasts of impact severity and likelihood (see Fig. 1), with warnings issued according to a traffic light scheme, ranging from Green to Red. Such an approach has been adopted in many places across the world^3,4,23^, and is currently being developed by Southeast Asian meteorological organisations in collaboration with the UK Met Office^24^.

The present studies focus on the ‘likelihood’ dimension of IBWs (rows in Fig. 1) and, specifically, the influence of impact severity information on this dimension. Rather than likelihood and severity being independent, as the orthogonal matrix implies, the aforementioned psychological research would predict that impact severity influences communications of impact likelihood, and thus the overall warning. Specifically, we assume that costs associated with under-communicating the likelihood of severe weather impacts (e.g. mitigations not being put in place) are extreme. Loss functions associated with over- versus under-estimates are therefore asymmetric for these impacts. Consequently, we predict that forecasters will issue warnings in a higher likelihood category on the WMO’s risk matrix for severe (versus minor) impacts with the same likelihood of occurrence (Hypothesis 1; see Table 1 for all research questions and hypotheses).

**Table 1:** Overview of research questions, hypotheses and findings by country.

Study	Research question	Hypothesis	Results
Pilot	(1) Do we observe a severity effect (i.e. are the same likelihoods interpreted differently according to the severity of impacts to which they refer?	H_1_ Higher warnings will be issued for the same likelihood category when it refers to a significant or severe impact versus a minimal or minor one	
Main	(1) Do we observe a severity effect in a dynamic IBW paradigm? (Replication of pilot)	H_1_ Higher warnings will be issued for the same likelihood category when it refers to a severe impact versus when it refers to a minor one	
	(2) Is the severity effect more pronounced in sequential IBW scenarios, rather than single warning IBW scenarios?	H_2_ The magnitude of the severity effect will be more pronounced at the second stage of a multi-stage forecasting scenario	
	(3) Does a severity effect have a continued influence when making sequential weather warnings?	H_3_ Likelihood warnings provided for identical events (in terms of likelihood and anticipated impact) 24 h from the event will be higher where the three-day forecast referred to a more severe impact	
	(4) Does a severity effect manifest itself when translating weather warnings to numerical likelihoods?	H_4_ Higher numerical likelihoods will be assigned to VPEs when these refer to a severe impact compared to a minor impact	
	(5) Are forecasters aware of the severity effect and what are their intuitions regarding its optimality?	Due to the exploratory nature of RQ_5_, we did not have specific predictions regarding this research question	Little evidence thus far

= Indonesia,  = Malaysia,  = Philippines,  = Vietnam.

The pilot study only included participants from Indonesia and the Philippines. Coloured flags without a strikethrough indicate support for hypotheses.

*Hypotheses relate to our intended manipulations of severity, in line with the countries’ impact tables. When investigating severity as perceived by participants (henceforward, always termed Perceived severity), a significant positive relationship between Perceived severity and perceived likelihood was observed in all countries.

**Figure 2:**
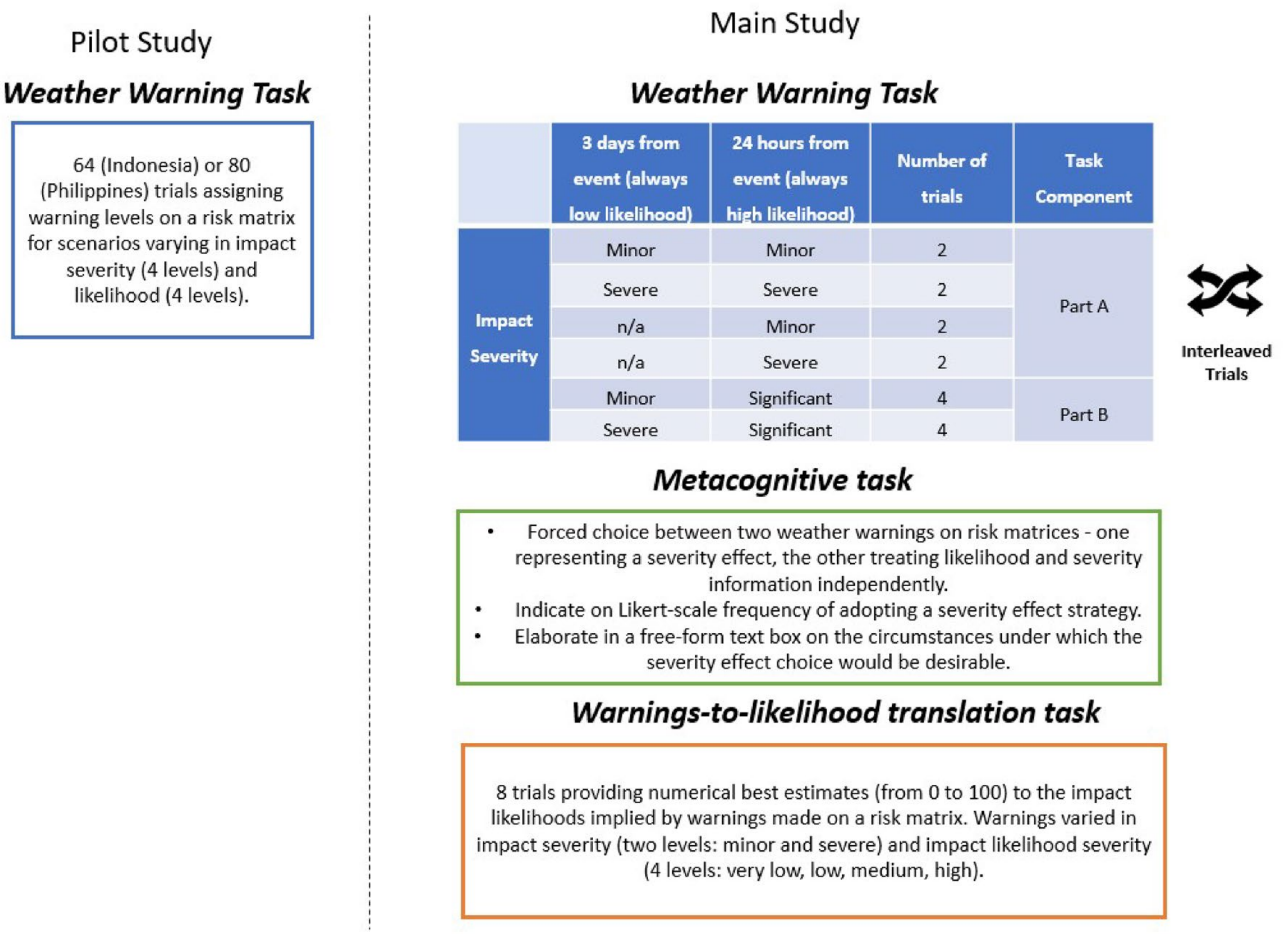
Methodological overview of the trials and tasks included in the Pilot study and Main study (for more detail see “Methods” section).

Given the dynamic nature of risk assessments and communications in weather forecasting, in the Main study we investigate: (a) how a *dynamic* weather forecasting environment impacts the Severity effect, and (b) potential downstream influences of the Severity effect within a dynamic weather forecasting scenario (see Fig. 2 for an overview of our methodology). For (a), we investigate whether making two forecasts about an event with a severe impact (3 days [Time 1] and one day [Time 2] from its occurrence) will have an additive effect, increasing the overall Severity effect (Hypothesis 2). For (b), a Severity effect at Time 1 is predicted to exert a continued influence at Time 2, even where information has changed to downgrade the severity of the event’s impact (Hypothesis 3). Such a continued influence would lead forecasts about the same anticipated event at Time 2 to differ as a function of what the available evidence *had* suggested at Time 1. Losee et al.^25^ showed that high-severity hurricane warnings had a continued influence on severity expectations, even when initial warnings were downgraded. Here, we test whether expected impact severity can influence *likelihood* communications in the same way. Finally, we probe communicators’ awareness and justifications for a Severity effect.

The present studies aim to increase our understanding of the prevalence and consequences of the Severity effect, within an applied weather forecasting context. In addition to implications in the weather domain, this research is of relevance to any domain where risk communications combine severity and likelihood considerations.

## Pilot study

We tested whether a Severity effect would be observed for categorical weather warnings in a within-participants design. To test Hypothesis 1, participants were presented with the numerical likelihood of a specific impact and asked to indicate a cell of the risk matrix corresponding to the warning they would issue (see Fig. 1). This tested whether a Severity effect occurs even when participants have a chance to express severity information in their communications (through the column response). Notably, this is typically not the case in previous research where participants were solely asked for a likelihood communication (e.g. Refs.^5,11^), whereby their only means of encouraging action would be to inflate likelihood estimates. Our research questions, hypotheses, methods and analyses were pre-registered (https://osf.io/rkz7v/?view_only=33ee4617e8914b99961eb60778c38d71 [Pilot Study—Study 3; Main study – Study 4; Studies 1 & 2 are unrelated and have been published^26,27^.

### Results

#### Planned analyses

**Figure 3:**
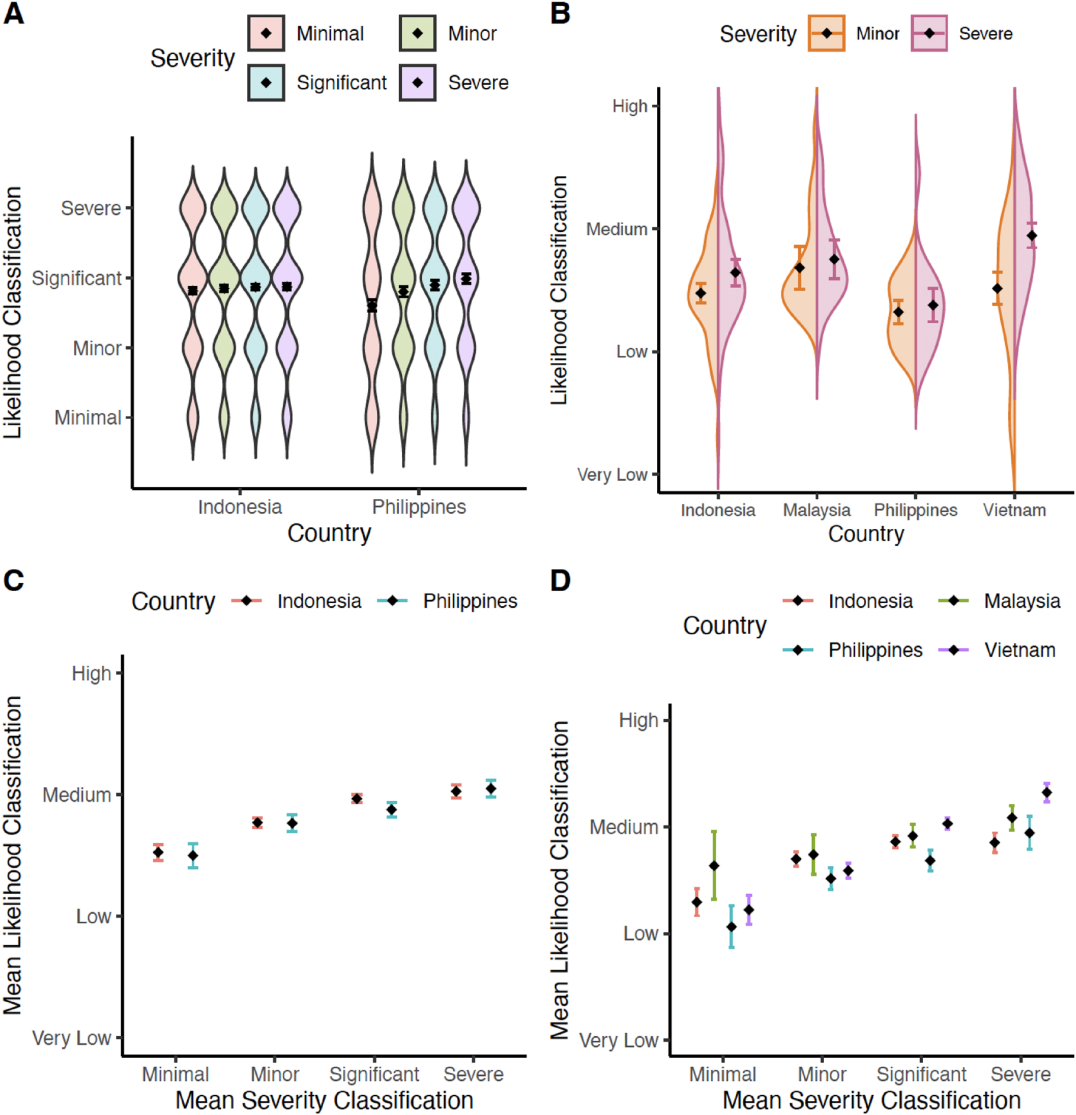
The top panel shows the distribution of likelihood classifications by manipulated severity category within each country in the (**A**) Pilot study and (**B**) Main study. The bottom panel shows the relationship between participants’ perceived severity classifications and their likelihood classifications within each country in the (**C**) Pilot study, and (**D**) Main study. Diamond = mean; Error bars = 95% CI of mean.

Responses obtained from the warning task were initially re-coded into the primary dependent variable of interest, representing the row (‘Likelihood rating’) the warning was on (Very Low—1, Low—2, Medium—3, High—4). Figure 3A suggests the presence of a Severity effect within the Philippines – higher likelihood classifications as impact severity increases – but not within Indonesia. This pattern was corroborated by a linear mixed-effect model (LMM), predicting likelihood classifications with three predictors: ‘Country’, ‘Severity’ and ‘Likelihood’ – the latter two variables representing the actual severity and likelihood levels provided in scenarios (all model specifications and complete outputs are included in Supplementary Information, SI). The apparent interaction between Severity and Country was confirmed, *F* (3, 9274.6) = 27.02, *p* < .0001, *η*_p_^2^ = 0.008% (see SI.1 for full model output). Tukey HSD corrected post-hoc comparisons confirmed the pattern suggested in Fig. 3A. A consistent Severity effect was observed in the Philippines sample (*p* < .03 for all pairwise Severity contrasts), but not in the Indonesian sample (all *p*s > .05; note that our analysis deviated from the pre-registered analysis, which was not fit to answer our research questions, as it did not account for our within-subjects design).

We also replicated the analysis carried out on our aggregated data on the individual country datasets. This confirmed the presence of a Severity effect in the Philippines but not Indonesia (see SI.2).

All LLMs were run using *R*^28^ in *R* Studio v 2022.12.0 + 353^29^ utilising the package *lme4* v 1.1–32^30^. In all analyses, we generated *p*-values via Satterthwaite’s method. Post-hoc comparisons for all LLM’s were conducted using *emmeans v 1.8.1–1*^31^ in *R Studio.*

#### Exploratory analyses

As a (non pre-registered) manipulation check, column (Perceived severity) responses were coded similarly to row responses (Minimal—1, Minor—2, Significant—3, Severe—4) and, as predicted, more severe impacts received more severe classifications in both countries, *F* (3, 136.8) = 145.52, *p* < .001, *η*_p_^2^ = 76%. This effect was, however, qualified by an interaction with Country, *F* (3, 136.8) = 34.32, *p* < .001, *η*_p_^2^ = 43% with a larger effect of Severity in the Philippines than Indonesia (see SI.3 for full descriptive and inferential statistics). Given this interaction, could the difference in the Severity effects between the two countries be a product of differences in participants’ *subjective severity* classifications? To address this, we tested for a relationship between perceived severity and likelihood classifications. Figure 3C suggests a positive relationship (reflecting a Severity effect) in both countries. Indeed, a LMM [Perceived likelihood ~ Perceived severity*Country + (1| ID)] revealed a main effect of Perceived severity, *F* (3, 9752) = 87.82, *p* < .001, *η*_p_^2^ = 3%, which was not moderated by Country (interaction: *F* [3, 9752] = 1.17, *p* = .32, *η*_p_^2^ = 0.005%). There was also no main effect of Country (*F* < 1). For full descriptive and inferential statistics see SI.4.

## Main study

The Main study addressed five research questions (Table 1) within a dynamic forecasting scenario that required forecasters to provide warnings at different lead times (3 days and 24 h; see Fig. 1) from a hydro-meteorological weather event.

### Results

To promote clarity, analyses will be presented for each research question in turn. Additional exploratory analyses are presented in SI.7. As some participants did not complete the entire survey, notes specify where analyses had missing datapoints. Responses obtained from the Main study’s primary task were re-coded as in the Pilot study. Note that the analyses we employed to address H_1_, H_2_ and H_3_ (LMMs) diverged from those pre-registered (mixed-ANOVAs). We changed our analysis plan for consistency with the pilot study, as well as the analyses addressing RQ4. Nonetheless, we replicated the findings reported in this paper for H_1_, H_2_ and H_3_ with the pre-registered analyses.

#### Replicating the ‘severity effect’ (H1)

We initially averaged each participant’s Likelihood ratings for all (ten) scenarios featuring severe impacts found in the weather warning task, regardless of whether they were three days or 24 h from the event (both Part A and Part B of the task – see Fig. 2) to obtain a ‘severe severity score,’ and computed a ‘minor severity score’ with the minor severity impacts in the same way. Figure 3B suggests the presence of a Severity effect, with higher likelihood classifications for severe impacts than minor impacts. Our analysis (see SI.5 for full output) confirmed a significant Severity effect, *F* (1, 206.8) = 42.05, *p* < .001, *η*_p_^2^ = 17%, but this was qualified by a Severity × Country interaction, *F* (3, 207.1) = 12.55, *p* < .001, *η*_p_^2^ = 15%.

Post-hoc pairwise comparisons (Tukey HSD corrected) confirmed a significant Severity effect in Indonesia, *t* (212) = 4.3, *p* < 0.001, and Vietnam, *t* (212) = 10.19, *p* < .001, but not in Malaysia, *t* (210) = 0.93, *p* =.35, or the Philippines, *t* (210) = 0.89, *p* = .37, partially supporting H_1_. We computed a ‘Severity effect score’ (see Figure B in SI.5.1 for distribution) as the difference between the sum of Likelihood ratings in severe and minor severity scenarios. Positive Severity effect scores conceptually demonstrate a participant used the Severity effect strategy at some point during the primary task. The percentage of participants who had a positive Severity effect score was: 61% in Indonesia, 52% in Malaysia, 47% in the Philippines, and 79% in Vietnam.

##### Exploratory analyses (H1)

Severity effects were not moderated by participants’ amount of experience with IBF and risk matrices (see SI.13). Severity classifications were more severe for severe impact trials than minor impact trials, *F* (1, 207.21) = 274.46, *p* < .001, *η*_p_^2^ = 57%, although this effect was again moderated by Country, *F* (3, 207.61) = 9.49, *p* < .001, *η*_p_^2^ = 12% (see SI.7 for full details). As in the Pilot study, we tested the relationship between participants’ severity classifications (Perceived severity) and their likelihood classifications. Figure 3D suggests a positive relationship (reflecting a Severity effect) in all countries. Although there was a Perceived severity × Country interaction, *F* (9, 5902.8) = 4.31, *p* < .001, *η*_p_^2^ = 0.6%, crucially there was a significant positive relationship between Perceived severity and Perceived likelihood classifications in all countries (all *p*s < .003; for full details see SI.8).

#### The severity effect in sequential versus single warning forecasting scenarios (H2)

This analysis utilised data from the second time point of sequential scenarios (24 h lead time – hereafter referred to as “T2”) included in *Part A* of the primary task (see Fig. 2). A ‘severe severity score’ and ‘minor severity score’ were again computed (Figure E, SI.9; there were no missing data points in the data utilised in this analysis. Due to a slight violation of the assumption of normality, as a robustness check, we carried out a paired-samples Wilcoxon Signed-Ranks Test to verify our main findings [see SI.9]).

Our LMM analysis included ‘Scenario Type’ as a factor (whether participants made a single warning 24 h from the event, or two warnings – one at three days from the event and one at 24 h from the event). Crucially (for H2), the Severity × Scenario Type interaction was not significant, *F* (1, 423.5) 2.11, *p* = .15, *η*_p_^2^ = 0.04%, suggesting that the Severity effect was not enhanced at the second stage of a multi-stage forecasting scenario, rather than in a single warning scenario, in contrast to H2. For full details see SI.9.

#### Continued influence of the severity effect (H3)

**Figure 4:**
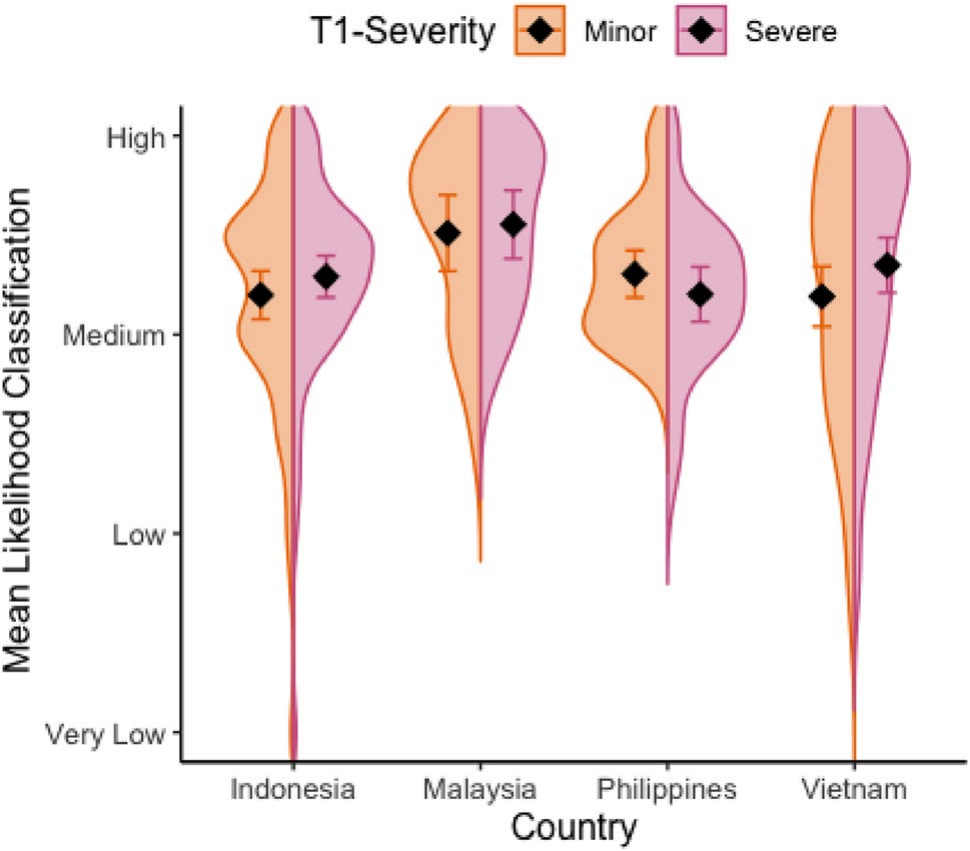
Distribution of likelihood classifications at T2 for severe and minor impact T1-severity scenarios, within each country. Diamond = mean; Error bars = 95% CI of mean.

This analysis used data obtained from the second time point of scenarios (T2) included in *Part B* of the primary task (see Fig. 2). Inspection of Fig. 4 suggests a potential continued influence of the Severity effect within Indonesia and Vietnam. Inferential analyses revealed no main effect of T1-Severity on warning likelihoods at T2, *F* (1, 208.17) = 3.24, *p* = .073; *η*_p_^2^ = 2%, but a significant Country × T1-Severity interaction was found, *F* (3,208.31) = 4.77, *p* = .003; *η*_p_^2^ = 6% (see SI.10 for full details). Follow-up tests revealed (consistent with H1) that the effect of T1-Severity was significant in Indonesia, *t* (214) = 2.76, *p* = .006, and Vietnam, *t* (214) = 3.82, *p* < .001, but not Malaysia, *t* (213) = 0.67,* p* = .50, or the Philippines, *t* (213) =  − 1.85, *p* = .07.

##### Exploratory analysis (H3)

We tested the relationship between participants’ *Perceived* severity classifications at T1, and their likelihood classifications at T2 (for full analysis output see SI.10.1). Replicating the results for manipulated severity (planned analysis), there was a main effect of Perceived T1-severity on likelihood classifications, *F* (3, 1602) = 4.45, *p* < .004, *η*_*p*_^*2*^ = 0.08%, which was moderated by a significant Perceived T1-severity × Country interaction, *F* (9, 1586.7) = 2.55, *p* = .007, *η*_*p*_^*2*^ = 0.01%. As with manipulated severity, a Continued influence effect was observed in Indonesia and Vietnam (*ps* < .04), but not Malaysia or the Philippines (see SI.10.1 for further details).

#### The severity effect when translating warnings to numerical likelihoods (H4)

**Figure 5:**
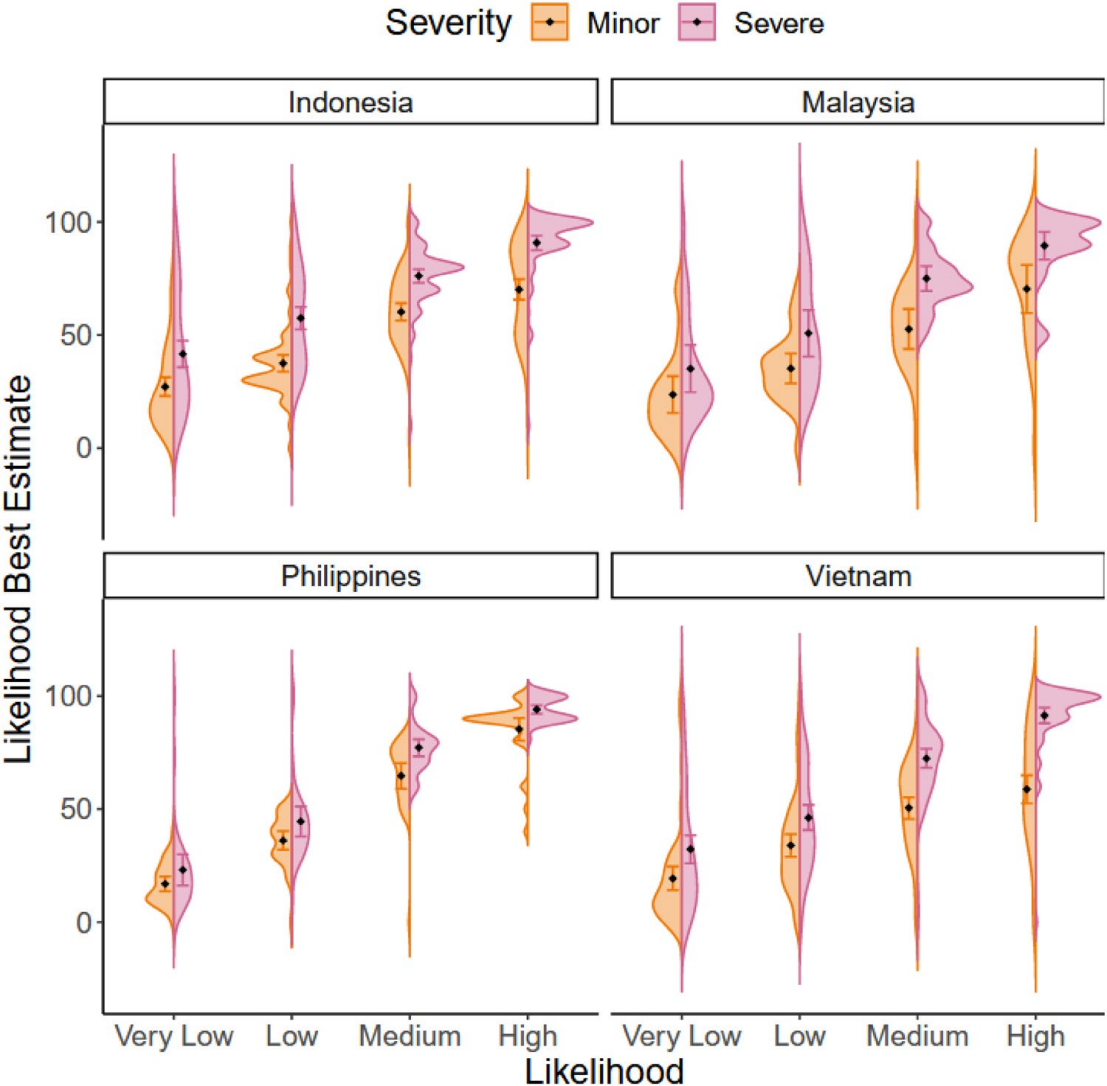
Distribution of best estimate likelihood ratings for events with minor and severe impacts of different likelihoods, within each country. Diamond = mean; Error bars = 95% CI of mean.

This analysis used data obtained from the warnings-to-likelihood translation task (see Fig. 2) – for full model output, see SI.11. Figure 5 shows that participants’ best estimates increased as the likelihoods implied by the warnings increased, supported by a significant effect of Likelihood, *F* (3, 288.67) = 236.44, *p* < .001, *η*_p_^2^ = 71%. Figure 5 also demonstrates higher numerical estimates for more severe impacts, *F* (1, 213) = 117.11, *p* < .001,* η*_p_^2^ = 35%.

In addition to the main effects, all four interactions were significant: Severity × Likelihood, *F* (3, 852) = 8.04, *p* < .001,* η*_p_^2^ = 3%; Severity × Country, *F* (3, 213) = 2.66, *p* = .049, *η*_p_^2^ = 4%; Likelihood × Country, *F* (9, 288.67) = 2.44, *p* = .011, *η*_p_^2^ = 7%; Severity × Likelihood × Country, *F* (9, 852) = 4.06, *p* < .001, *η*_p_^2^ = 4%, as well as the main effect of Country, *F* (3, 194.02) = 7.13, *p* < .001,* η*_p_^2^ = 10%. Although a Severity effect was observed at each level of Likelihood (all *p*s < .001), follow-up tests of the three-way interaction demonstrated that these effects were not consistent across all four countries, despite the consistent numerical trends (Fig. 5). Tukey HSD follow-up tests revealed a Severity effect at each level of likelihood within Indonesia and Vietnam (for all comparisons, *p* < .001), no Severity effect at any likelihood level in the Philippines (for all comparisons *p* > .05), and finally a Severity effect at a ‘medium’ and ‘high’ likelihood level in Malaysia (*p*s < .005). These findings therefore partially support H4.

#### Metacognition (RQ5)

These analyses used data from the Metacognitive task (Fig. 2). Given the exploratory nature of RQ5, we primarily focus on descriptive statistics.

Choosing Risk Matrix B in the binary choice question represented explicitly endorsing a Severity effect strategy (see Fig. 2). This Severity effect strategy was only chosen by a minority of participants (Indonesia 19%, Malaysia 17%, Philippines 19%, Vietnam 32%). As such, despite the findings we reported in SI.5.1 illustrating that most participants demonstrated a Severity effect in the primary task (though not significantly so in Malaysia or the Philippines), when asked to overtly choose between a Severity effect and ‘non-Severity effect’ strategy, most participants in each country chose a non-Severity effect strategy.

**Figure 6:**
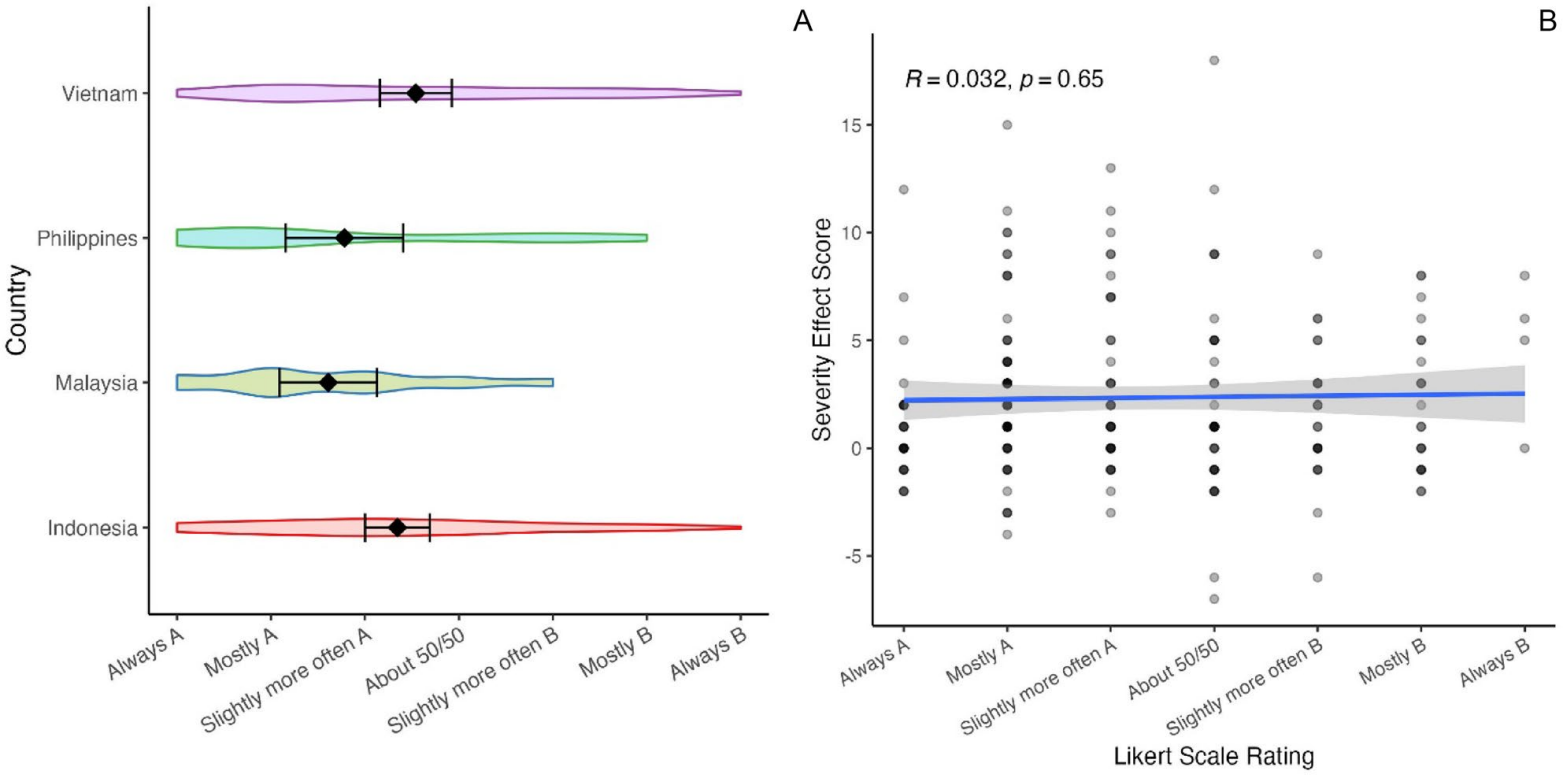
(**A**) Distribution of Likert scale rating of participants in each country. Diamond = mean rating. Error bars = 95% CI of the mean. Always A (− 3) represents always choosing a non-Severity effect strategy, and Always B (+ 3) represents always choosing a Severity effect strategy. (**B**) Scatterplot of participant Likert-scale ratings and Severity effect score (severe impact Severity score – minor impact Severity score) in the aggregated sample with Spearman-rho’s correlation coefficient.

The above results do not, however, necessarily demonstrate an inconsistency between participants’ IBWs and their explicit metacognition about those decisions. The fact that, across 20 judgments, participants show some evidence for a Severity effect, does not mean that they would typically choose to escalate likelihood classifications in the majority of instances. Indeed, turning to the Likert scale ratings, only 23/179 participants reported that they would always choose Risk matrix A over Risk matrix B, potentially suggesting that sometimes they would choose B. Most participants report ‘mostly adopting’, or ‘slightly more often adopting’, a non-Severity effect strategy (A) than a Severity effect strategy (B; see Fig. 6A).

Is there any consistency between participants’ self-reported likelihood of engaging in a Severity effect communication strategy (Likert scale ratings) and their actual communications (Severity effect score: severe impact Severity score – minor impact Severity score)? Although noisy, Fig. 6B suggests not. Indeed, there is no significant relationship between participants’ Likert scale responses and their Severity effect scores *r* (204) = 0.032, *p* = .65 (Spearman’s rank correlation). See SI.12 for additional analyses.

Analyses relating to the qualitative responses associated with RQ5 can be seen in SI.12.1. In summary, consistent with the binary responses, the majority of participants (71%) reported that they would never use Risk Matrix B for this IBW, although some participants did mention considerations associated with severity and preparedness/precautionary actions.

## Discussion

Across two studies, we found that perceptions of impact severity influenced likelihood classifications when communicating IBWs, in both a static (single-warning) and dynamic (multiple-warning) decision-making context. The within-participants design enabled us to detail that, overall, the majority of participants provided responses consistent with a Severity effect. The observed interactions with Country demonstrated that the manipulation of severity did not have the same influence across all countries. Tentatively, this lack of a direct effect of severity on likelihood classifications in Malaysia and the Philippines may reflect the fact that the survey was not presented in these participants’ first language. Nonetheless, there was a significant positive relationship between participants’ perceived severity classifications and likelihood classifications in all countries for both studies. This result is suggestive evidence for a dependence between likelihood and severity information when making IBWs – diverging from the implied independence asserted in the WMO’s guidelines^3,4^. This is a particularly pertinent finding in light of the ongoing move towards implementing IBW across the globe using such a risk matrix approach and, indeed, the usage of such matrices in other contexts^32–34^. The observed relationship between likelihood and severity is also consistent with prior research on forecaster impact perceptions, in which the two factors co-varied positively^26^.

Our results are consistent with Severity effects previously observed in the psychological literature, with higher interpretations of VPEs which refer to a severe versus neutral outcome^5–9^. We demonstrate here that this (same) effect occurs in the opposite direction. That is, numerical likelihoods describing more severe impacts are communicated with higher likelihood categories on a verbal scale (ranging from ‘very low’ to ‘high’ as per the WMO’s risk matrix).

We further extend previous research on the Severity effect, by noting its presence when participants have the means to provide a response via the use of a two-dimensional risk matrix. This is in contrast to methods of risk communication that only require a likelihood estimate^5^. Utilising the latter method, if one wanted to emphasise the severity of an impact, one could *only* do so by inflating the likelihood. By providing a risk matrix to our participants, however, we gave them the option to communicate about both severity and likelihood independently. Observing a Severity effect under these conditions highlights the robustness of this effect. One caveat to this is that participants might have wanted to provide different coloured warnings for impacts of different severity (for instance to promote action), and there are some locations on the risk matrix which would require them to increase likelihood classifications as well as severity classifications in order to do so. Analyses presented in SI.6 demonstrate that this was not a key driver of the observed Severity effect.

The final part of the main study required forecasters to act as communication recipients and translate the likelihoods implied by warnings made on the risk matrix to numbers. We found some evidence that higher numerical estimates were provided for warnings of severe impacts, even when these shared the same likelihood as minor severity impacts, consistent with previous research on the translation of VPEs (e.g. ‘unlikely’; Refs.^5–9^). These findings suggest that a Severity effect remains present when interpreting the warnings made by “communicators.” Furthermore, within this context, participants are seemingly not correcting for a Severity effect in warnings, even though these are the same participants who themselves inflated IBW likelihoods for severe impacts. Such a result is indicative of a considerably influential effect: whilst impact severity increases the categorical communication of likelihood, these categorical communications, in turn, are interpreted as denoting higher numerical likelihoods.

### The consequences of the severity effect

Previous studies exploring the Severity effect have typically focused on judgments at a single time point, neglecting the dynamic nature of the environment in which these judgments are typically made (see Ref.^19^ for a notable exception). In real-world forecasting situations, multiple warnings might be made for the same event, at different lead times. We therefore included scenarios in our study that required forecasters to make IBWs for the same event when this was initially three days away and, subsequently, one day away. Providing an additional warning for a weather event at three days’ lead time did not amplify the Severity effect at a one-day lead time. We did, however, observe a continued influence of the Severity effect in Indonesia and Vietnam, such that higher warnings were provided for impacts of the same severity and likelihood (at a one-day lead time) if they had been preceded by a three day warning for a severe, rather than minor impact. The latter result might reflect a recognition that the initial (three day) forecast likely still carries some diagnostic weight and therefore the ‘medium severity’ one-day forecast might be expected to be of higher severity where the three day forecast was for severe (versus minor) impacts.

We strongly caution against evaluative judgments of forecaster proficiency following the identification of a Severity effect in this manuscript. We hold that, in an ideal world, perfectly-calibrated probabilistic forecasts (i.e. that do not show a Severity effect) are most beneficial (see also Ref.^35^). Weather warnings (and other risk communications) are provided to a variety of different consumers. Different consumers will have different propensities for taking action, and thus the inflation of likelihood communications for severe events will likely be beneficial for some, and detrimental for others. Maintaining the independence of severity and likelihood enables recipients to combine the information to inform actions consistent with their own decision thresholds. Such a discussion, however, disregards the potential to calibrate forecasts. As Rothfusz et al.^35^ highlight, a perfectly calibrated probabilistic forecast is likely an unrealistic goal. Without perfect calibration, a sensitivity to asymmetric loss functions (e.g. through demonstration of a Severity effect) has been shown to be a rational response to uncertainty surrounding likelihood estimates (Refs.^36,37^, see also Ref.^13^). In the context of IBW, this can protect against under-warnings that could lead to a failure to take a threat seriously enough and thus failure to engage in appropriate mitigative actions, with potentially fatal consequences. The less confident a forecaster is in a probabilistic forecast product, the more they should adjust their forecast in response to an asymmetric loss function. Note that, in many situations, forecasters will not have defined probabilistic information provided to them. In these situations, we might expect the influence of severity to be greater, due to greater uncertainty around the probabilities. The degree to which forecasters’ sensitivity to asymmetric loss functions is sensitive to probabilistic uncertainty is an important topic for future research.

Our results demonstrate that likelihood estimates for severe impacts are higher than those for less severe impacts. In line with other work on the Severity effect, we are silent on whether estimates of severe events are less or more accurate than those of less severe events. Attempts to mitigate the effect must therefore consider whether it is more desirable to reduce the communicated likelihood of severe impacts or increase that of non-severe impacts. Ultimately, whether communicators decide that the Severity effect should be celebrated or eradicated, knowledge of the underlying processes associated with decision-making is important for enhancing transparency, knowledge exchange and consistency in risk analysis and communication.

### Summary, further considerations and future directions

The current studies demonstrated the non-independence of likelihood and severity with real-world information in the high stakes, applied context of IBW. We extended our investigation to explore the implications of this effect, revealing a potential persistence of Severity effects in sequential forecasting paradigms. Furthermore, we observed the presence of Severity effects in later stages of interpretation within the IBW framework, particularly when converting warnings made by others on a risk matrix, into numerical likelihoods. We additionally presented initial evidence to indicate that, although the presence of this Severity effect strategy was abundant across the overall sample, forecasters were seemingly unaware of utilising such a strategy at the time they were making IBWs (RQ5). We should note here, though, that RQ5 was something of an auxiliary Research Question in the current project, and materials were not optimised to test metacognition (see e.g. Ref.^38^). Further research is thus necessary before firm conclusions can be drawn here.

Although we have noted that the observed Severity effect is consistent with an asymmetric loss function account^11,12^, the psychological mechanism underlying the effect requires further testing. Previous demonstrations of the Severity effect when translating VPEs to numbers have noted the importance of controlling for the base rate of the events under investigation^7,19^. In the current context, events with severe impacts are (thankfully) rarer than minor impacts. The choice of higher likelihood classifications for the former might therefore reflect a pragmatic communication strategy (i.e. a 40% likelihood is ‘likely’ if compared with a base rate of 20%, but ‘unlikely’ if compared with a base rate of 60%). Whilst possible (and seemingly sensible), it should be made clear how these likelihood classifications are intended to be used and understood. Note that recent (unpublished) work from our research group^39^ suggests that the Severity effect in translations of numbers to VPEs holds in a weather warning context even where the base rate is empirically controlled for by manipulating the consequences of the same event about which the likelihood is asked (following^5^).

In line with the pragmatics associated with an IBW, warnings issued using the risk matrix (Fig. 1) consist of a single ‘tick’ in a single cell of the matrix. In reality, multiple cells might be appropriate. A weather front might be approaching with a high likelihood of minor impacts, and a lower likelihood of significant impacts. What warning should the forecaster issue? Whilst multiple ticks within the matrix would enable the disambiguation of the communication, currently forecasters must use their judgment to issue the warning that they perceive as the most informative and useful. How forecasters do this is an important question for future research, but the increase in likelihood classifications as perceived severity increased (Fig. 3C,D) suggests that this is not a key underlying explanation for the current results.

The generality of the observed effect across different meteorological agencies and other risk assessment contexts should be explored. If the current effect does reflect forecasters’ sensitivity to asymmetric loss functions, it is important to note that this will not always predict an increased likelihood communication for more severe outcomes. The expense and disruption associated with issuing a red warning (see Fig. 1), for example, might shift the direction of the asymmetry, such that forecasters err in the direction of under-forecasting likelihood where a red warning is at stake.

The current paradigm involved the assignment of a warning based on information about one impact. In reality, the contexts in which we make decisions are far richer. Hazards will often, for example, cause multiple impacts varying in features including severity and duration. The current research represents a significant starting point to the investigation of the psychological processes underlying the integration of likelihood and severity information in complex risk assessment and communication. Future research simulating real-world contexts as closely as possible will further increase the generalisability of our results. As reasoning with information on severity and likelihood is not limited to weather forecasting contexts, these findings have ramifications for any domain where decisions are made on the basis of risk assessments, including medical diagnosis and intelligence analysis.

Overall, our findings demonstrate the strength and continued influence of Severity effects. Recruiting weather professionals, we replicated previous findings showing that increased severity led to increased likelihood communications and show that this occurs not only when translating words to numerical expressions, but even when translating numerical expressions to words. The fact that the Severity effect still occurs (and is not corrected for) when the same communicators act as recipients further underlines its pervasiveness.

## Methods pilot study

### Participants

Forecasters were recruited in Indonesia (via Badan Meteorologi, Klimatologi, dan Geofisika [BMKG], the Indonesian meteorological agency and the Indonesian State College Of Meteorology Climatology And Geophysics [STMKG]) and the Philippines (via Philippine Atmospheric, Geophysical and Astronomical Services Administration [PAGASA, the Filipino meteorological agency) across a two-week period. A total of 150 participants completed the study (for full demographic details of each sample, see Table 2). Participation in the study was not remunerated. Informed consent was obtained from all participants. Ethical approval for both studies reported was granted from the Departmental Ethics Chair for Experimental Psychology (University College London), approval number: EP/2020/008. All methods were carried out in accordance with relevant guidelines and regulations, including the Declaration of Helsinki.

**Table 2:** Demographic details of samples in each country for the pilot study and the main study^1^.

	Country (partner)	Data collection period^2^	Sample, size	Demographics	IBF experience	Risk matrix experience	Forecasting experience
Pilot study	Indonesia (BMKG)	01/03/2021–19/03/2021	113	77 male, 32 female, 4 prefer not to say, aged between 18 and 42 (Mdn = 21)	28.3% little or no experience; 30.1% some training; 38.9% some experience; 2.7%, a lot of experience	n/a	n/a
	Philippines (PAGASA)	01/03/2021–19/03/2021	37	21 male, 16 female, aged between 25 and 62 (Mdn = 35)	29.7% little or no experience; 48.7% some training; 21.6% some experience; 0% a lot of experience	n/a	n/a
Main study	Indonesia	12/12/2021–28/01/2022	84	n/a	1.2% had never heard of it; 22.6% had heard of it; 16.7% received training on it; 25% use it occasionally; 34.5% use it regularly	7% had never seen it before; 31% had seen it before but not used it; 26.2% received training on it; 35.7% use it in their work/studies	77.4% prepare forecasts; 19% use forecasts; 3.6% do neither of the above
	Malaysia	27/11/2021–28/01/2022	23	n/a	4.3% had never heard of it; 30.4% had heard of it; 17.4% received training on it; 26.1% use it occasionally; 21.7% use it regularly	21.7% had never seen it before; 47.8% had seen it before but not used it; 17.4% had received training on it; 13% use it in their work/studies	65.2% prepare forecasts; 13% use forecasts; 21% do neither of the above
	Philippines	18/11/2021–28/01/2022	32	n/a	3.1% had never heard of it; 25% had heard of it; 34.4% received training on it; 34.4% use it occasionally; 3% use it regularly	9.4% had never seen it before; 25% had seen it before but not used it; 56.3% had received training on it; 9.4% use it in their work/studies	37.5% prepare forecasts; 34.4% use forecasts; 28.1% do neither of the above
	Vietnam	12/12/2021–28/01/2022	74	n/a	5.4% had never heard of it; 33.8% had heard of it; 20.3% received training on it; 21.6% use it occasionally; 18.9% use it regularly	13.5% had never seen it before; 31.1% had seen it before but not used it; 8.1% had received training on it; 47.3% use it in their work/studies	75.7% prepare forecasts; 24.3% use forecasts

^1^In the main study, demographic information such as age and gender was not obtained from participants. This decision was made due to the (in our opinion, more informative) variables related to forecasting experience, IBF experience and experience using risk matrices (which we pre-registered and included in exploratory analyses).

^2^In the pre-registration of the Main study, we stated that as many responses as possible would be gathered within the timeframe of one calendar month (dates from December 18th until January 2nd were not included in this calculation), with a minimum of 20 forecasters required to complete the study within each country^40^. However, at the end of the given timeframe of one calendar month of data collection in the Philippines and Malaysia, we had not reached the stated minimum number of forecasters required to complete the study. For this reason, we extended the data collection period in these two countries until the end of the one-calendar-month data collection period of the remaining two countries (Indonesia and Vietnam) which began at a later date. For data collection periods see Table 2. We undertook no data analysis until the decision had been made to collect no further data.

### Design and materials

The design of the study was based on the WMO’s^3^ impact risk matrix for impact-based weather warnings (see Fig. 1). Likelihood (4 levels – high, medium, low, very low) and impact severity (4 levels – minimal, minor, significant, and severe) were manipulated within-participants. (In the Philippines, impact severity was presented with the following four levels – minimal/very low, minor/low, significant/medium, severe/high). Participants were presented with the (numerical) likelihood information of a specific impact and asked to indicate a cell of the impact matrix, corresponding to the warning they would issue.

Impacts were presented in the context of a specific hazard – heavy rainfall, selected in collaboration with the Southeast Asian authors on the basis of its high relevance to their countries and the existence of a pre-developed impact table (see SI.14, Table Q and R). following discussions with PAGASA, in the Philippines, the decision was made to present additional rainfall information corresponding to PAGASA’s warning thresholds (green – less than 7.5 mm; yellow – 7.5–15 mm; orange – 15–30 mm, red – 30 mm). We used these numbers to supplement the impact manipulation, such that minimal impacts were presented with the lowest amount of rainfall, minor impacts with 7.5–15 mm, and so on. Materials were presented in English in the Philippines and translated into Bahasa in Indonesia. The translation was undertaken by SA and RN and checked by AW, with random selections additionally checked by RB and SJ.

In Indonesia (the Philippines), participants were presented with a total of 64 (80) forecasts, made up of four (five) impacts from each of the four categories of severity, accompanied by each of the four likelihood levels. To ensure that participants paid attention and to prevent task fatigue, we varied the numerical probabilities which accompanied the impacts: ‘very low’ = 10–30% or 20–40%; ‘low’ = 30–50% or 40–60%; ‘medium’ = 60–80% or 70–90% and high = 80–100%. This also served to introduce some additional ambiguity into the task, given that forecasters could not just use the translation as prescribed in Ref.^41^: 10–30% (very low); 30–50% (low); 50–80% (medium) and > 80% (high). The specific impacts were selected from those which had the highest level of (forecaster) agreement of warning level with the original impact categorisation in the impact tables in Ref.^27^. Owing to a programming error in the version of the study sent to the Philippines, three participants saw both numerical conditions for the very low, low, medium conditions for Impacts 12, 13, 14 and 15 (e.g. seeing both 10–30% likelihood and 20–40% likelihood). In these instances, we took the answer which they saw first. For Impact 17, all participants saw both numerical versions for the low condition, so we took the answer which they saw first (30–50% likelihood). We replicated all analyses using a Philippines dataset that (i) removed these three participants, and (ii) removed Impact 17 as a whole. Results were unchanged from when we included the full Philippines sample as included in the main analysis.

### Procedure

This online study was run using Qualtrics, with participants able to complete the study in more than one session. Before beginning the tasks, participants were asked a series of demographic questions. They were asked to indicate gender (male/female/prefer not to say); age; indicate if, in their work, they typically use forecasts/or prepare them; their level of experience with IBF (little or no experience/some training/some experience/a lot of experience).

Participants were then presented with instructions for the task, asking them to consider the presented impact and likelihood information, and indicate where they would place the forecast within the WMO impact risk matrix. They were asked to consider the impacts solely in relation to their specific country.

On the next screen, participants were presented with one of the impacts and a specified likelihood level and asked to indicate a cell on the impact matrix, corresponding to the warning they would issue. The impacts were presented in a random order to avoid order effects. The subsequent screen showed another randomly presented impact and likelihood level, and so on and so forth, until the participant had given a warning for the full set of forecasts. Finally, participants were thanked and debriefed.

## Methods main study

### Participants

Participants were recruited in Indonesia, Malaysia, the Philippines, and Vietnam (through BMKG, STMKG, MET Malaysia, PAGASA, and the National Center for Hydro-Meteorological Forecasting [NCHMF] respectively). A total of 213 participants took part in the study (for demographic details, see Table 2). Participation in the study was voluntary and was not remunerated. In the Philippines, there was an administrative error in the distribution of the online Main study survey (such that the recruitment email [SI.16] included our experimental research questions).

### Design and materials

The central element of the online study required participants to make an IBW using WMO’s Impact Risk Matrix (see Fig. 1). All manipulations were within-participant, and the primary dependent variable of interest was the matrix row (i.e. likelihood) of participants’ assigned warnings. The study was programmed using Qualtrics (www.qualtrics.com) and comprised of two main components (hereafter referred to as ‘primary task’ and ‘secondary tasks’), with the primary task being further sub-divided into two parts (hereafter referred to as ‘Part A’ and ‘Part B’). Part A and Part B of the primary task were interleaved together, and this distinction would not have been apparent to participants. For clarity, we will describe each component of the study in turn – highlighting how it relates to our research questions.

#### Primary task: Part A

Part A of the primary task adopted a 2 (impact severity: minor, severe) × 2 (scenario type: single warning, sequential warnings) design and primarily addressed Research Questions 1 and 2. In this part of the task, we included eight forecasting scenarios. Four of these scenarios required participants to provide a single weather warning on the risk matrix when a hypothetical weather event was 24 h away and there was either a high likelihood of a severe impact (in two scenarios) or a high likelihood of a minor impact (in two scenarios). The other four scenarios required participants to make two weather warnings on the risk matrix sequentially; one when the hypothetical weather event was three days away, and one when the same weather event was 24 h away.

In all four sequential forecasting scenarios, the likelihood of the given impact was low at the first stage (3 days from the weather event) and high at the second stage (24 h from the weather event) – representing the increased certainty a forecaster would typically have, closer to the event. However, in two of these scenarios the severity of the impact was minor at both temporal stages, and in the other two scenarios the severity of the impact was severe at both temporal stages. In this part of the task, we counterbalanced across participants which specific impacts (described further at the end of the Methods section) appeared in scenarios eliciting only one weather warning 24 h from the event, and which appeared in scenarios eliciting two weather warnings (three days from the event and 24 h from the event).

#### Primary task: Part B

Part B of the primary task addressed Research Question 3. We included eight sequential forecasting scenarios. In four of the sequential scenarios – across the two temporal stages (three days from, and 24 h from, a hypothetical weather hazard) – the likelihood of the impact changed from low to high and the severity from minor to significant. In the other four sequential scenarios, the likelihood changed from low to high and the severity from severe to significant. As such, in this part of the task, our manipulation was the severity of the impact at the first forecasting stage (minor or severe; ‘T1-severity’; see Fig. 2 for a breakdown of the number of scenarios we included in the primary task broken down by combination of severity, likelihood, and warning period [three days from event or 24 h from event]).

Throughout the entire study, to ensure participants remain engaged, and to increase the ambiguity of some scenarios, we varied the numerical probabilities which accompanied the impacts, such that a ‘low’ likelihood was either described as 10–30% or 20–40% and a ‘high’ likelihood was described as either 60–80% or 70–90%. This also prevented participants from simply using the likelihood translations prescribed by the UK Met Office^41^, if they knew of them: 10–30% (very low); 30–50% (low); 50–80% (medium) and > 80% (high).

#### Secondary tasks

The secondary tasks addressed Research Questions 4 and 5. In the first part of the secondary task (addressing Research Question 5), participants were presented with a single scenario depicting a weather warning made on a risk matrix for a minor impact with low likelihood. Through a forced-choice question, participants were required to choose which one of two warning choices they would make for a severe impact of the same low likelihood: (A) a warning choice which assigns a warning that increases on the severity column of the risk matrix, but not the likelihood row, or (B) one that assigns a higher likelihood warning to the severe impact event than the original likelihood implies.

Participants were additionally asked about the likelihood of employing strategies represented by each warning choice in situations such as the one described in the forced-choice question. The 7-point Likert scale ranged from ‘Always A’ (− 3) to ‘Always B’ (3), with the middle (0) representing choosing warning choices A and B approximately 50% of the time each. Finally, we included a question requiring participants to explain, using a free-form text box, the reasons for why, and the circumstances under which, warning choice B might be employed as a strategy.

In the second part of the secondary task (addressing Research Question 4), we included eight forecasting scenarios in which participants were asked to provide a numerical best estimate (in the form of a percentage) for the impact likelihoods implied by a weather warning as indicated on a risk matrix. In these eight scenarios, warnings represented different likelihoods and severities of impacts (2 impact severity: minor or severe × 4 impact likelihood: very low, low, medium, and high.

#### Impacts used in the scenarios

All impacts included in the scenarios described a single specific hazard of high consequence for the specific country. Surveys distributed in the Philippines, Indonesia, and Vietnam related to ‘heavy rainfall.’ The survey distributed in Malaysia related to ‘river flooding.’ Events were localised to Metro Manila in the Filipino survey, Hanoi in the Vietnamese survey, Jakarta in the Indonesian survey, and Kelantan in the Malaysian study.

In the scenarios included in each survey we selected impacts from the in-country impact tables for the appropriate hazard that had a clear escalation from minor to severe, and that represented a variety of different impact domains (e.g. infrastructure, property damage, health and sanitation). For surveys describing weather events relating to heavy rainfall, the decision was made to present additional rainfall information corresponding to the country’s forecasting service’s warning thresholds. Thresholds were agreed upon by each in-country partner as the following: minor impact was associated with 7.5–15 mm rainfall; significant impact with 15–30 mm, and severe impact with 30 mm or more. The impacts chosen for each partner country can be viewed in SI.15, Tables S–U. In the version of the study to be administered to forecasters in Vietnam, we utilised the same impact table as that administered to forecasters in the Philippines. This was necessary because, at the time we distributed the survey, Vietnam was in the process of transitioning to impact-based weather forecasting practices and developing its impact tables.

#### Language

Materials for Malaysia and The Philippines were presented to participants in English. Indonesian and Vietnamese materials were translated into Bahasa (by SA and RN), and Vietnamese (by HN and DTQ), respectively. These language choices reflect the comfort of the different nations in working in the different languages (and English is the primary working language of PAGASA).

### Procedure

The study was run using Qualtrics (www.qualtrics.com), with participants being asked to complete the study in a single session. At the start of the survey, participants were asked a series of demographic questions, eliciting information on the profession, and experience with impact-based weather forecasting, which was asked was asked using a forced-choice question with the following options: (i) I have never heard of it [impact-based weather forecasting], (ii) I have heard of it, (iii) I have received training on it (iv) I have used it occasionally (in either work or training exercises) and (v) I use it regularly in my work/studies. More specifically, experience with WMO’s risk matrices was also measured, using a forced-choice question with the following options: (i) I have never seen it before, (ii) I have seen it before but not used it (iii) I have received training on it and (iv) I use it in my work/studies.

Participants were then presented with instructions for the primary task, asking them to consider the presented impact and likelihood information, and indicate the warning they would issue, by clicking in an appropriate cell of the WMO Impact Risk Matrix (see Fig. 1) at either one (24 h from) or two (three days from and 24 h from) stages of a weather event. Before beginning the primary task, participants were shown an example trial and had a chance to review the set of instructions.

Once they began the primary task, participants completed the 16 scenarios comprising both Part A and Part B (see Fig. 2) in a randomised order (an example *sequential* scenario is shown in Fig. 1. After completing the primary task, participants were given instructions on the secondary tasks and proceeded to complete them. Here, they firstly answered questions relating to evaluating the weather warning choices made by an imagined colleague for impacts of different severities but the same likelihood of occurrence using forced-choice, Likert-scale, and free-form textbox questions (described in more detail in the Design and Materials section of the Primary Study Methods). In the second part of the secondary tasks, they were required to provide the best estimate likelihood estimates for six weather warning choices varying in both impact severity and verbal likelihood expressions (presented in randomised order). Finally, participants were thanked for their participation and debriefed as to the aims of the study.

### Supplementary Information

Supplementary Information.

## Data Availability

All materials and data are available at: https://osf.io/rkz7v/?view_only=33ee4617e8914b99961eb60778c38d71.

## References

[1] Von Neumann J, Morgenstern O (1947). Theory of Games and Economic Behavior.

[2] Savage LJ, Savage LJ (1954). The foundations of statistics. The Foundations of Statistics.

[3] World Meteorological Organization (2015). WMO Guidelines on Multi-hazard Impact-based Forecast and Warning Services.

[4] World Meteorological Organization, ‘WMO Guidelines on Multi-hazard Impact-based Forecast and Warning Services - Part II: Putting Multi-hazard IBFWS into Practice’, Geneva, Switzerland. https://reliefweb.int/report/world/wmo-guidelines-multi-hazard-impact-based-forecast-and-warning-services-part-ii-putting (Accessed 8 January 2022) (2021).

[5] Harris AJL, Corner A (2011). Communicating environmental risks: Clarifying the severity effect in interpretations of verbal probability expressions. J. Exp. Psychol. Learn. Mem. Cognit..

[6] Bonnefon J-F, Villejoubert G (2006). Tactful or doubtful?: Expectations of politeness explain the severity bias in the interpretation of probability phrases. Psychol. Sci..

[7] Pepper S, Prytulak LS (1974). Sometimes frequently means seldom: Context effects in the interpretation of quantitative expressions. J. Res. Personal..

[8] Weber EU, Hilton DJ (1990). Contextual effects in the interpretations of probability words: perceived base rate and severity of events. J. Exp. Psychol. Human Percept. Perform..

[9] Villejoubert G, Almond L, Alison L (2009). Interpreting claims in offender profiles: the role of probability phrases, base-rates and perceived dangerousness. Appl. Cogn. Psychol..

[10] Sirota M, Juanchich M (2012). To what extent do politeness expectations shape risk perception? Even numerical probabilities are under their spell!. Acta Psychol..

[11] Harris AJL, Corner A, Hahn U (2009). Estimating the probability of negative events. Cognition.

[12] Weber EU (1994). From subjective probabilities to decision weights: The effect of asymmetric loss functions on the evaluation of uncertain outcomes and events. Psychol. Bull..

[13] Batchelor R, Peel DA (1998). Rationality testing under asymmetric loss. Econ. Lett..

[14] Goodwin P (1996). Statistical correction of judgmental point forecasts and decisions. Omega.

[15] Granger CWJ (1969). Prediction with a generalized cost of error function. J. Oper. Res. Soc..

[16] Dhami MK, Mandel DR (2021). Words or numbers? Communicating probability in intelligence analysis. Am. Psychol..

[17] Mastrandrea, M. D. et al. Guidance Note for Lead Authors of the IPCC Fifth Assessment Report on Consistent Treatment of Uncertainties’, Intergovernmental Panel on Climate Change, (2010).

[18] Juanchich M, Sirota M (2013). Do people really say it is “likely” when they believe it is only “possible”? Effect of politeness on risk communication. Q. J. Exp. Psychol..

[19] Holtgraves T, Perdew A (2016). Politeness and the communication of uncertainty. Cognition.

[20] Parmar S, Thomas RP (2020). Effects of probabilistic risk situation awareness tool (RSAT) on aeronautical weather-hazard decision making. Front. Psychol..

[21] United Nations Office for Disaster Risk Reduction, ‘The human cost of disasters: an overview of the last 20 years (2000-2019)’, United Nations, 10.18356/79b92774-en (2020).

[22] Jha S (2018). Natural disasters, public spending, and creative destruction: A case study of the Philippines. SSRN Electron. J..

[23] Harrowsmith M (2020). The future of forecasts: Impact-based forecasting for early action. Red Cross Red Crescent Clim. Cent..

[24] Beckett R, Hartley A (2020). Progress on the Development of Impact Based Forecasting in South East Asia.

[25] Losee JE, Naufel KZ, Locker L, Webster GD (2017). Weather warning uncertainty: High severity influences judgment bias. Weather Clim. Soc..

[26] Jenkins SC (2022). Impact-based forecasting in South East Asia—What underlies impact perceptions?. Int. J. Disaster Risk Reduct..

[27] Jenkins SC (2022). Investigating the decision thresholds for impact-based warnings in South East Asia. Int. J. Disaster Risk Reduct..

[28] R Core Team, ‘R: A language and environment for statistical computing.’ Vienna, Austria. https://www.r-project.org/ (Accessed 27 April 2022) (2022).

[29] Posit Team (2022). RStudio: Integrated Development Environment for R.

[30] Bates D, Mächler M, Bolker BM, Walker SC (2015). fitting linear mixed-effects models using lme4. J. Stat. Softw..

[31] Lenth, R. V. et al. emmeans: Estimated marginal means, aka least-squares means. https://CRAN.R-project.org/package=emmeans (Accessed 17 October 2022) (2022).

[32] World Economic Forum, ‘The Global Risks Report 2021’, Geneva, Switzerland. https://www.weforum.org/reports/the-global-risks-report-2021 (Accessed 11 May 2021) (2021).

[33] UK Cabinet Office, ‘National Risk Register: 2020 Edition. https://www.gov.uk/government/publications/national-risk-register-2020 (Accessed 10 May 2023) (2020).

[34] International Organization for Standardization, ‘IEC 31010:2019. Risk management—Risk assessment techniques’. https://www.iso.org/standard/72140.html (Accessed 10 May 2023) (2019).

[35] Rothfusz LP (2018). FACETs: A proposed next-generation paradigm for high-impact weather forecasting. Bull. Am. Meteorol. Soc..

[36] Whiteley L, Sahani M (2008). Implicit knowledge of visual uncertainty guides decisions with asymmetric outcomes. J. Vis..

[37] Lawrence M, O’Connor M (2005). Judgmental forecasting in the presence of loss functions. Int. J. Forecast..

[38] Newell BR, Shanks DR (2014). Unconscious influences on decision making: A critical review. Behav. Brain Sci..

[39] Harris, A. J. L., Kau, S. H. & Liefgreen, A. Likelihood increases with communication: The severity effect in a communication chain (2024).

[40] Simmons JP, Nelson LD, Simonsohn U (2011). False-positive psychology: Undisclosed flexibility in data collection and analysis allows presenting anything as significant. Psychol. Sci..

[41] UK Met Office (2020). The National Severe Weather Warning Service Best Practice Guide.

