# Supplementary material for: Severity influences categorical likelihood communications: A case study with Southeast Asian weather forecasters
Source: Sci Rep. 2024 Jun 25;14:14607. doi: 10.1038/s41598-024-64399-5 (PMC11199697; doi:10.1038/s41598-024-64399-5)
Supplement: Supplementary file 1 — Supplementary Information. [file 41598_2024_64399_MOESM1_ESM.docx]

**Supplementary Information**

NB. In the analyses presented in this document, “Severity” or “Sev.” refers to manipulated severity of the impacts, unless specifically labelled “Perceived severity” or “Perceived Sev.” – in which case it refers to participants’ perceived impact severity obtained through their classifications on the risk matrix.

**Contents**

Table of Contents

[Section 1: Pilot Study, Aggregated Model Specification and Output 2](#_Toc166070656)

[Section 2: Pilot Study, Analyses Within Each Country 3](#_Toc166070657)

[2.1. Philippines 3](#_Toc166070658)

[2.2. Indonesia 4](#_Toc166070659)

[2.3. Between-Country Post-hoc Comparisons 4](#_Toc166070660)

[Section 3: Pilot Study, Exploratory Analysis: Perceived Severity as Outcome Variable 5](#_Toc166070661)

[Section 4: Pilot Study, Exploratory Analysis: Perceived Severity as Predictor 7](#_Toc166070662)

[Section 5: Main Study, Output RQ1 Analysis 9](#_Toc166070663)

[5.1. Main Study: Distribution of Individual Severity Effect Scores (RQ1 Additional Analysis) 10](#_Toc166070664)

[Section 6: Main Study Exploratory Analysis: Cell Color Change Analysis 11](#_Toc166070665)

[Section 7. Main Study Exploratory Analysis: Severity Classifications as Outcome 13](#_Toc166070666)

[Section 8. Main Study, Exploratory Analysis: Perceived Likelihood By Perceived Severity 16](#_Toc166070667)

[Section 9: Main Study, Output RQ2 Analysis 18](#_Toc166070668)

[Section 10: Main Study, RQ3 Analysis 21](#_Toc166070669)

[10.1: Exploratory Analyses H3 22](#_Toc166070670)

[Section 11: Main Study, Output RQ4 Analysis 24](#_Toc166070671)

[Section 12: Main Study, RQ5 Exploratory Analyses 26](#_Toc166070672)

[12.1. Main Study, RQ5 Qualitative Data Analysis 28](#_Toc166070673)

[Section 13: Main Study Exploratory Analyses 30](#_Toc166070674)

[13.1 Professional Experience And The Severity Effect 30](#_Toc166070675)

[13.2. Experience With IBF and the Severity Effect 31](#_Toc166070676)

[13.2. Experience With Risk Matrices and the Severity Effect 32](#_Toc166070677)

[Section 14: Pilot Study Impact Tables 34](#_Toc166070678)

[Section 15: Main Study Impact Tables 35](#_Toc166070679)

[Section 16: Main Study Recruitment Email to Philippines 38](#_Toc166070680)

# Section 1: Pilot Study, Aggregated Model Specification and Output

The model we built was: Perceived likelihood ~ Country + Severity*Likelihood + Country* Severity + (Likelihood| ID). The model’s explanatory power related to the fixed effects alone (marginal *R*^2^) was 0.45. The full output of the analysis is shown in Table A.

**Table A.**

*Output of Analysis of model: Perceived likelihood ~ Country + Severity*Likelihood + Country* Severity + (Likelihood| ID)*

| **Perceived likelihood** | | | |
| --- | --- | --- | --- |
| *Predictors* | *Estimates* | *CI* | *p* |
| (Intercept) | 1.85 | 1.75 – 1.95 | **< .001** |
| Country [Philippines] | -0.21 | -0.31 – -0.12 | **< .001** |
| Severity [minor] | 0.11 | 0.04 – 0.18 | **.003** |
| Severity [significant] | 0.10 | 0.03 – 0.17 | **.007** |
| Severity [severe] | 0.08 | 0.01 – 0.15 | **.023** |
| Likelihood [low] | 0.74 | 0.65 – 0.83 | **< .001** |
| Likelihood [medium] | 1.39 | 1.26 – 1.52 | **<. 001** |
| Likelihood [high] | 1.71 | 1.56 – 1.86 | **< .001** |
| Country [Philippines] *Severity [minor] | 0.16 | 0.09 – 0.23 | **< .001** |
| Country [Philippines] *Severity [significant] | 0.24 | 0.16 – 0.31 | **<. 001** |
| Country [Philippines] *Severity [severe] | 0.33 | 0.25 – 0.40 | **< .001** |
| Severity [minor] *Likelihood [low] | -0.09 | -0.19 – 0.00 | .059 |
| Severity [significant] *Likelihood [low] | -0.06 | -0.15 – 0.04 | .245 |
| Severity [severe] *Likelihood [low] | -0.05 | -0.15 – 0.04 | .260 |
| Severity [minor] * Likelihood [medium] | -0.11 | -0.20 – -0.01 | **.023** |
| Severity [significant] * Likelihood [medium] | -0.09 | -0.19 – 0.00 | .054 |

| Severity [severe] * Likelihood [medium] | -0.09 | -0.18 – 0.01 | .073 |
| --- | --- | --- | --- |
| Severity [minor] * Likelihood [high] | -0.07 | -0.16 – 0.02 | .146 |
| Severity [significant] * Likelihood [high] | -0.01 | -0.10 – 0.08 | .835 |
| Severity [severe] * Likelihood [high] | 0.03 | -0.06 – 0.13 | .488 |
| **Random Effects** |  |  |  |
| σ2 | 0.36 |  |  |
| τ00 ResponseId | 0.26 |  |  |
| τ11 ResponseId.likelihoodlow | 0.14 |  |  |
| τ11 ResponseId.likelihoodmedium | 0.47 |  |  |
| τ11 ResponseId.likelihoodhigh | 0.73 |  |  |
| ρ01 | -0.83 |  |  |
|  | -0.88 |  |  |
|  | -0.90 |  |  |
| ICC | 0.32 |  |  |
| N ResponseId | 149 |  |  |
| Observations | 9864 |  |  |
| Marginal R^2^ / Conditional R^2^ | 0.447 / 0.623 |  |  |

# Section 2: Pilot Study, Analyses Within Each Country

## Philippines

We fitted a linear mixed model (Perceived likelihood ~ Severity*Likelihood + (Likelihood| ID) to predict participants’ likelihood classifications with two predictors: ‘Severity’ and ‘Likelihood’. The model’s explanatory power related to the fixed effects alone (marginal *R*^2^) was 0.46. Our analysis revealed that the main effect of ‘Severity’ is statistically significant and small, *F* (3,2653.2) = 50.35, *p* < .001, *ηp2* = 5%, the main effect of ‘Likelihood’ is statistically significant and large, *F* (3, 32.8) = 50.85, *p* < .001, *η ^2^* = 82%, and the interaction between ‘Severity’ and ‘Likelihood’ is not statistically significant and very small, *F* (9, 2653.2) = 0.87,

*p*

*p* = .55, *η ^2^* = 0.002 %. For post-hoc comparisons on differences in likelihood classifications by scenario severity, averaged over scenario likelihood, see Table B.

*p*

**Table B.**

*Pairwise comparisons with Tukey HSD correction.*

| Severity Contrast | Estimate | SE | df | t.ratio | *p*.value |
| --- | --- | --- | --- | --- | --- |
| Minimal - Minor | -0.2 | 0.03 | 2669 | -6.13 | <.0001 |
| Minimal - Significant | -0.29 | 0.03 | 2670 | -8.9 | <.0001 |
| Minimal - Severe | -0.38 | 0.03 | 2670 | -11.6 | <.0001 |
| Minor - Significant | -0.09 | 0.03 | 2670 | -2.86 | .022 |
| Minor - Severe | -0.18 | 0.03 | 2669 | -5.55 | <.0001 |
| Significant - Severe | -0.09 | 0.03 | 2669 | -2.69 | .036 |

*N.B. results averaged over Likelihood level.*

## Indonesia

We fitted a linear mixed model (Perceived likelihood ~ Severity*Likelihood + (Likelihood| ID) to predict participants’ likelihood classifications with two predictors: ‘Severity’ and ‘Likelihood’. The model’s explanatory power related to the fixed effects alone (marginal *R*^2^) was 0.55. Our analysis revealed that the main effect of ‘Severity’ is not statistically significant and small, *F* (3,171.8) = 1.72, *p* = .16, *η ^2^* = 3%, the main effect of ‘Likelihood’ is statistically significant and large, *F* (3, 113.2) = 140.31, *p* < .001, *η ^2^* = 79%, and the interaction between ‘Severity’ and ‘Likelihood’ is not statistically significant and very small, *F* (9, 6405.4) = 1.18, *p* = .3, *η ^2^* = 0.0016 %.

*p*

*p*

*p*

## Between-Country Post-hoc Comparisons

Post-hoc comparisons of our main analysis on aggregated data showed significant between- country differences in likelihood ratings between Indonesia and the Philippines for minimal severity scenarios (*p* = .0009), and severe scenarios (*p* = .018). As such, participants in our Philippines sample gave significantly lower likelihood ratings to minimal severity scenarios

compared to Indonesia, and significantly higher likelihood ratings to severe scenarios compared to Indonesia.

# Section 3: Pilot Study, Exploratory Analysis: Perceived Severity as Outcome Variable

The model we built was Perceived severity ~ Country * Severity + Likelihood * Country + (1

+ Severity | Response ID). The model’s explanatory power related to the fixed effects alone (marginal *R*^2^) was 0.24, conditional *R*^2^= 0.48. Our analysis (for full model output see Table C) found no main effect of Country, *F*(1,145.5) = 0.26, *p* = 0.6, a main effect of Severity, *F*(3, 136.8) = 145.52, *p* < .001, a main effect of Likelihood, *F* (3, 9274.9) = 159.5, *p* < .001, a significant interaction between Severity and Country, *F*(3, 136.8) = 34.32, *p* < .001, and a significant interaction of Country and Likelihood, *F*(3,9274.9) = 3.4, *p* = .017. Tukey-adjusted post-hoc comparisons revealed a significant difference in perceived severity ratings between all pairwise comparisons of severity levels (all *p*s < .001) in the Philippines (see Figure A for direction). In Indonesia, we found a significant difference in perceived severity ratings between all pairwise comparisons of severity levels (all *p*s < .001) except for between significant and severe levels (*p* = .9).

**Figure A.**

*Pilot Study: Mean severity classifications by severity levels in each country.*


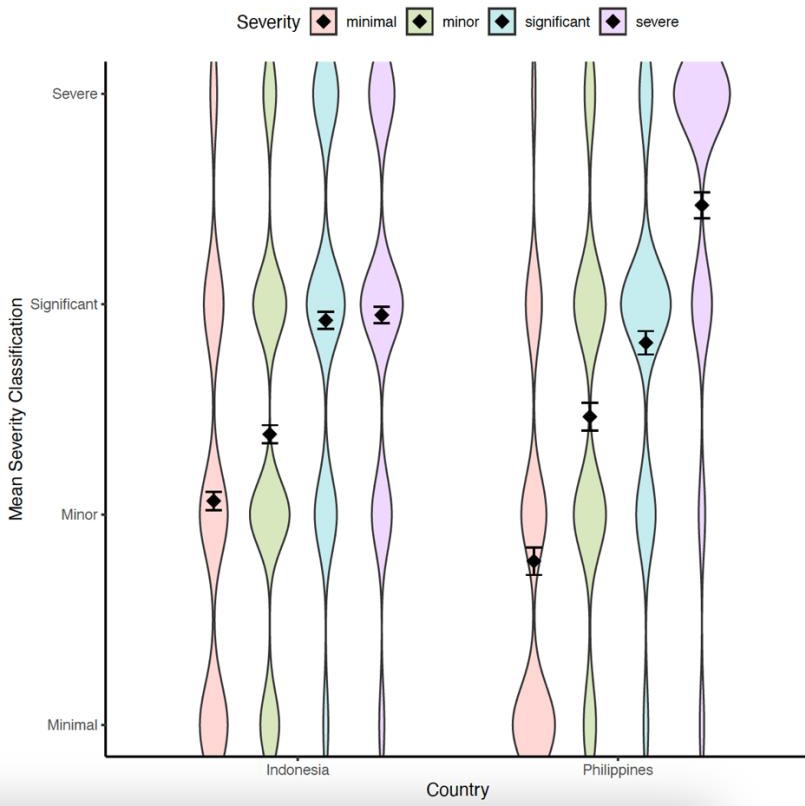


**Table C.**

*Full output of model: perceived severity ~ Country * severity + likelihood * Country + Model: (1 + severity | Response ID)*

| **Perceived severity** | | | |
| --- | --- | --- | --- |
| *Predictors* | *Estimates* | *CI* | *p* |
| (Intercept) | 1.79 | 1.69 – 1.90 | **< .001** |
| Country [Philippines] | -0.25 | -0.46 – -0.03 | **.025** |
| Severity [minor] | 0.31 | 0.24 – 0.39 | **< .001** |
| Severity [significant] | 0.86 | 0.74 – 0.97 | **< .001** |
| Severity [severe] | 0.88 | 0.76 – 1.00 | **< .001** |
| Likelihood [low] | 0.19 | 0.14 – 0.23 | **< .001** |

| Likelihood [medium] | 0.38 | 0.33 – 0.42 | **< .001** |
| --- | --- | --- | --- |
| Likelihood [high] | 0.53 | 0.48 – 0.57 | **< .001** |
| Country [Philippines] × Severity [minor] | 0.37 | 0.22 – 0.52 | **< .001** |
| Country [Philippines] × Severity [significant] | 0.18 | -0.05 – 0.41 | .134 |
| Country [Philippines] × Severity [severe] | 0.80 | 0.56 – 1.04 | **< .001** |
| Country [Philippines] × Likelihood [low] | -0.02 | -0.11 – 0.07 | .675 |
| Country [Philippines] × Likelihood [medium] | -0.05 | -0.14 – 0.04 | .278 |
| Country [Philippines] × Likelihood [high] | -0.13 | -0.22 – -0.04 | **.003** |
| **Random Effects** |  |  |  |
| σ2 | 0.51 |  |  |
| τ00 ResponseId | 0.28 |  |  |
| τ11 ResponseId.severityminor | 0.10 |  |  |
| τ11 ResponseId.severitysignificant | 0.32 |  |  |
| τ11 ResponseId.severitysevere | 0.36 |  |  |
| ρ01 | -0.47 |  |  |
|  | -0.66 |  |  |
|  | -0.64 |  |  |
| ICC | 0.31 |  |  |
| N ResponseId | 149 |  |  |
| Observations | 9864 |  |  |
| Marginal R^2^ / Conditional R^2^ | 0.243 / 0.481 |  |  |

# Section 4: Pilot Study, Exploratory Analysis: Perceived Severity as Predictor

The model we built was: Perceived likelihood ~ Perceived severity*Country + (1| ID). The model’s explanatory power related to the fixed effects alone (marginal *R*^2^) was 0.03 (conditional *R*^2^ = .07). Our analysis revealed a main effect of Perceived severity, *F* (3, 9752) = 87.82, *p* < .001, *ηp2*= 3%, which was not moderated by Country (interaction: *F* [3, 9752] =

1.17, *p* = .32, *η ^2^*= .04%). There was also no main effect of Country, *F* (1, 139.2) = 0.62, *p* =

*p*

.43, *η ^2^*= .4%. For full model output, see Table D.

*p*

**Table D.**

*Full output of model: Perceived likelihood ~ Perceived severity*Country + (1| ID).*

| **Perceived likelihood** | | | |
| --- | --- | --- | --- |
| *Predictors* | *Estimates* | *CI* | *p* |
| (Intercept) | 2.53 | 2.46 – 2.60 | **< .001** |
| Perceived severity [Minor] | 0.24 | 0.17 – 0.31 | **< .001** |
| Perceived severity [Significant] | 0.43 | 0.36 – 0.50 | **< .001** |
| Perceived severity [Severe] | 0.51 | 0.43 – 0.59 | **< .001** |
| Country [Philippines] | -0.05 | -0.18 – 0.08 | .436 |
| Perceived severity [Minor] × Country [Philippines] | 0.04 | -0.09 – 0.17 | .554 |
| Perceived severity [Significant] × Country [Philippines] | -0.04 | -0.16 – 0.09 | .560 |
| Perceived severity [Severe] × Country [Philippines] | 0.06 | -0.08 – 0.20 | .388 |
| **Random Effects** |  |  |  |
| σ2 | 0.88 |  |  |
| τ00 ResponseId | 0.04 |  |  |
| ICC | 0.04 |  |  |
| N ResponseId | 149 |  |  |
| Observations | 9864 |  |  |
| Marginal R^2^ / Conditional R^2^ | 0.033 / 0.075 |  |  |

# Section 5: Main Study, Output RQ1 Analysis

The model was: Total Score ∼ Severity * Country + (1| Participant). The model’s total explanatory power (conditional *R*^2^) was 0.72, and that related to fixed effects alone (marginal *R*^2^) was 0.17. The full output of the analysis can be seen in Table E.

**Table E.**

*Output of model: Total Score ∼ Severity * Country + (1| Participant).*

|  |  | **Total Score** |  |
| --- | --- | --- | --- |
| *Predictors* | *Estimates* | *CI* | *p* |
| (Intercept) | 24.71 | 23.78 – 25.64 | **< .001** |
| Severity [Severe] | 1.72 | 0.95 – 2.50 | **< .001** |
| Country [Malaysia] | 2.11 | 0.12 – 4.11 | **.038** |
| Country [Philippines] | -1.49 | -3.25 – 0.27 | .096 |
| Country [Vietnam] | 0.43 | -0.93 – 1.79 | .536 |
| Severity [Severe] — Country [Malaysia] | -1.03 | -2.67 – 0.61 | .219 |
| Severity [Severe] — Country [Philippines] | -1.16 | -2.61 – 0.29 | .117 |
| Severity [Severe] — Country [Vietnam] | 2.59 | 1.46 – 3.72 | **< .001** |
| **Random Effects** |  | |  |
| σ2 | 6.25 | |  |
| τ00 participant | 12.25 | |  |
| ICC | 0.66 | |  |
| N participant | 211 | |  |
| Observations | 417 | |  |
| Marginal R^2^ / Conditional R^2^ | 0.167 / 0.719 | |  |

Post-hoc pairwise comparisons (Tukey HSD corrected) demonstrated a significant difference in the average Likelihood ratings relating to severe impact scenarios between Indonesia and the Philippines (*p* = .01), Indonesia and Vietnam (*p* < .001), Malaysia and the Philippines (*p* = .009), and Philippines and Vietnam (*p* < .001). In addition, we found significant differences between the average Likelihood ratings associated with minor impact scenarios between Malaysia and the Philippines, *p* = .01. As such, the Philippines provided lower likelihood classifications in minor impact scenarios compared to Malaysia, and *lower* likelihood classification in severe impact scenarios compared to all other countries.

## Main Study: Distribution of Individual Severity Effect Scores (RQ1 Additional Analysis)

Figure B shows the distribution of severity effect scores in the whole sample.

**Figure B.**

*Histogram of severity scores in aggregated sample organised in bins of 5.*


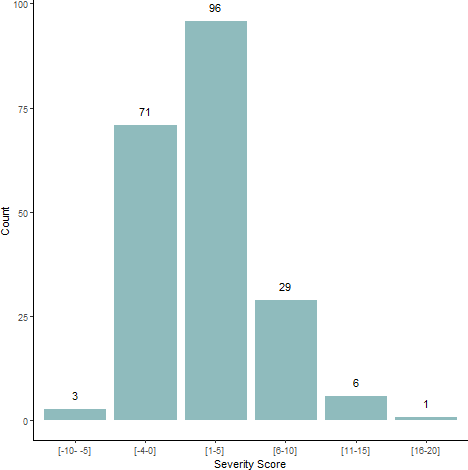


# Section 6: Main Study Exploratory Analysis: Cell Color Change Analysis

It is possible that the observed severity effect reflects an idiosyncrasy in the risk matrix, relating to the matching of colored warnings with severity and likelihood. There are some parts of the risk matrix (namely, cells [3,1] and [3,3]^[[1]](#footnote-1)^) where it is not possible to increase a warning’s color solely by increasing impact severity (moving horizontally in the matrix) – an increase in impact likelihood is also required. We wanted to ascertain whether the Severity effect we observed was entirely attributable to this color artefact.

Were the Severity effect entirely attributable to the color artefact, it will be driven solely by responses in [3,1] and [3,3] to minor impacts and [4,2] and [4,4]^[[2]](#footnote-2)^ to severe impacts (color increases from yellow (‘be aware’) to orange (‘be prepared’) and orange to red (‘take action’) respectively). Across the dataset, we regressed participants’ Severity effect scores (difference in their likelihood ratings for severe versus minor impacts) against the number of minor impact trials where they placed warnings in either of cells [3,1] and [3,3] and the number of severe impact trials where they placed warnings in either [4,2] or [4,4] as well as the interaction of these variables (which we term CellColorChangeMinor and CellColorChangeSevere respectively). If the color artefact is contributing to our results, the intercept of this model should be zero.

We fitted a linear model (estimated using OLS) to predict Severity Score with CellColorChangeMinor and CellColorChangeSevere. See Figure C for scatterplots of our variables of interest, and Table F for the number of classifications labelled as ‘CellColorChange’ for Severe impact and Minor impact trials, within each country. For full model output see Table G. The number of Severe warnings in these specific cells has a negative beta value (beta = -0.46, 95% CI [-0.80, -0.11], *t*(202) = -2.59, *p* = .010), which is not in line with the color artefact hypothesis. The interaction term is positive but not (quite) significant, (beta = 0.14, 95% CI [-0.00359, 0.28], *t*(202) = 1.92, *p* = .056) thus not providing statistically reliable evidence for the influence of the color artefact. Finally, the positive and significant intercept (2.47, 95% CI [1.52, 3.42], *t*(202) = 5.13, *p* < .001) demonstrates a significant severity effect is still predicted even in the absence of any responses in either of these specific cells.


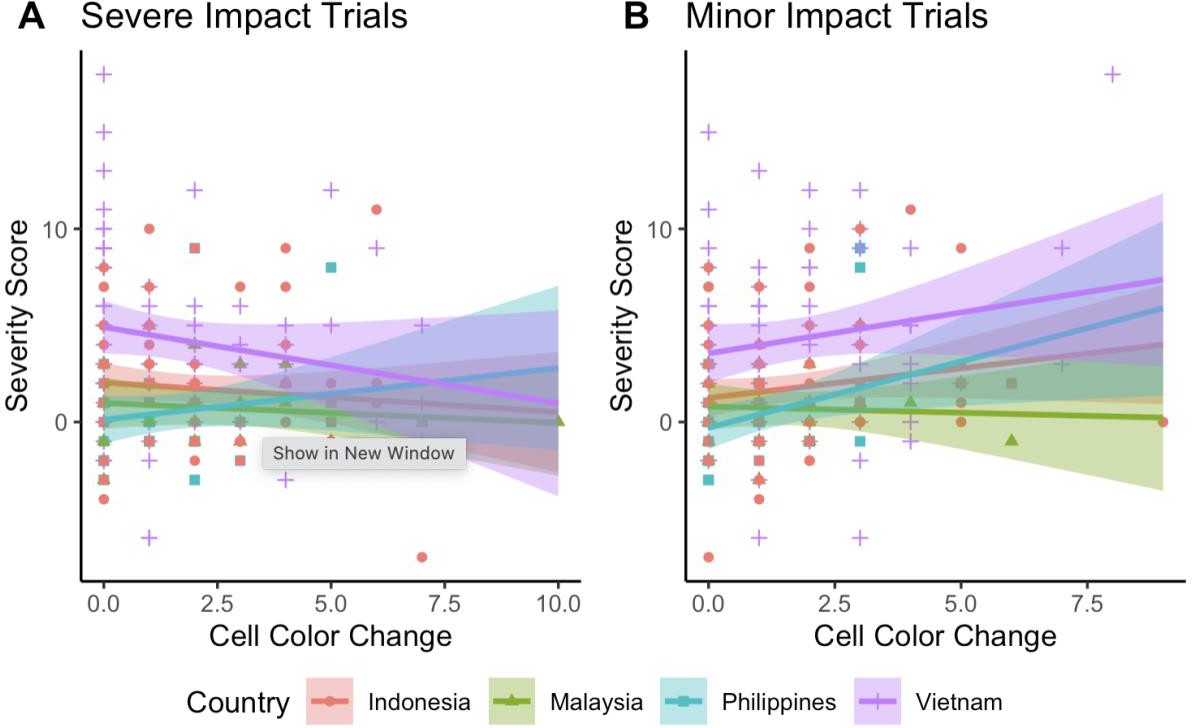


**Figure C.**

*Scatterplot of severity and cell color change scores, with regression lines, for each country in A) Severe impact trials and B) Minor impact trials.*

**Table F.**

*Number of classifications labelled as ‘CellColorChange’ for Severe impact and Minor impact trials, within each country – out of total number of trials in each manipulated severity category (%).*

| Country | Severity in Trials | CellColorChange N (%) |
| --- | --- | --- |
| Indonesia | Minor | 140 (16.7%) |
|  | Severe | 155 (18.5%) |
| Malaysia | Minor | 38 (16.5%) |
|  | Severe | 64 (27.8%) |
| Philippines | Minor | 40 (12.5%) |
|  | Severe | 53 (16.5%) |
| Vietnam | Minor | 130 (17.8%) |
|  | Severe | 110 (14.9%) |

**Table G.**

*Full model output for analysis: lm(Severity Score ~ CellColorChangeMinor* CellColorChangeSevere).*

|  | **Severity Score** | | |
| --- | --- | --- | --- |
| *Predictors* | *Estimates* | *CI* | *p* |
| (Intercept) | 2.47 | 1.52 – 3.42 | **<.001** |
| CellColorChangeMinor | 0.19 | -0.18 – 0.56 | .321 |
| CellColorChangeSevere | -0.46 | -0.80 – -0.11 | **.010** |
| CellColorChangeMinor × CellColorChangeSevere | 0.14 | -0.00 – 0.28 | .056 |
| Observations | 206 | | |
| R^2^ / R^2^ adjusted | 0.067 / 0.053 | | |

# Section 7. Main Study Exploratory Analysis: Severity Classifications as Outcome

We averaged each participant’s *Severity* ratings for all (ten) scenarios featuring high severity impacts found in the weather warning task, regardless of whether they were three days or 24 hours from the event (both Part A and Part B of the task) to obtain a ‘high severity score,’ and computed a ‘low severity score’ with the low severity impacts in the same way. We built a LMM: lmer(Total_Score ~ Severity*Country + (1|participant), where Total Score is the *total perceived severity score.* The model's total explanatory power was substantial (conditional R^2^

= 0.69) and the part related to the fixed effects alone (marginal R^2^) was 0.37.

Our analysis confirmed that severity classifications increased as manipulated severity increased, *F* (1, 207.21) = 274.46, *p* < .001, *η ^2^* = 57%. We also found a Severity × Country interaction, *F* (3, 207.61)) = 9.49, *p* < .001, *η ^2^* = 12%, and a main effect of Country, *F* (3, 210.17) = 7.27, *p* < .001, *ηp2* = 9%. Post-hoc pairwise comparisons showed a Severity effect on severity classifications within Indonesia, *t*(213) = -15.12, *p* < .001, Malaysia, *t* (210) = -3.1, *p* = .002, the Philippines, *t* (210) = -9.9, *p* < .001, and Vietnam, *t* (213) = -9.7, *p* < .001.

*p*

*p*

For graphical representation of severity classifications by severity level in each country, see Figure D. For full model output see Table H.


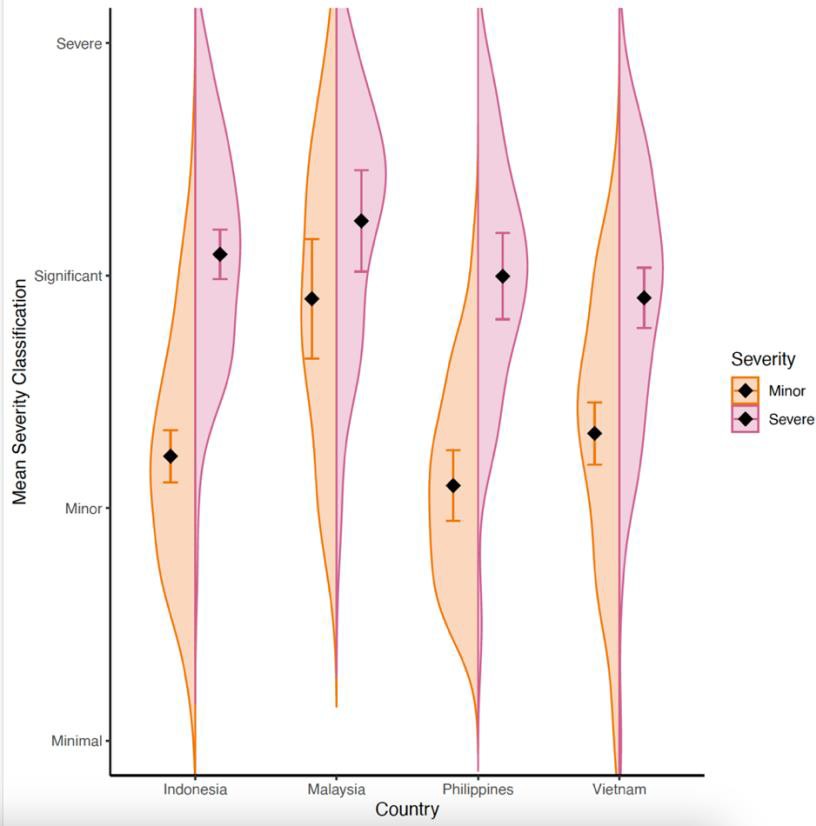


**Figure D.**

*Severity classifications by trial severity level within each country. Diamond = mean; Error bars = 95% CI of mean.*

**Table H.**

*Full model output for lmer(Total_Score ~ Severity*Country + (1|Participant)*

|  |  | **Total Score** |  |
| --- | --- | --- | --- |
| *Predictors* | *Estimates* | *CI* | *p* |
| (Intercept) | 22.26 | 21.14 – 23.38 | **< .001** |
| Severity [Severe] | 8.70 | 7.58 – 9.82 | **< .001** |
| Country [Malaysia] | 6.74 | 4.35 – 9.14 | **< .001** |
| Country [Philippines] | -1.29 | -3.40 – 0.83 | .232 |
| Country [Vietnam] | 0.87 | -0.77 – 2.52 | .296 |
| Severity [Severe] ×Country [Malaysia] | -5.35 | -7.73 – -2.97 | **< .001** |

| Severity [Severe] ×Country [Philippines] | 0.30 | -1.80 – 2.40 | .777 |
| --- | --- | --- | --- |
| Severity [Severe] ×Country [Vietnam] | -2.79 | -4.42 – -1.15 | **.001** |
| **Random Effects** |  |  |  |
| σ2 | 13.09 |  |  |
| τ00 participant | 13.61 |  |  |
| ICC | 0.51 |  |  |
| N participant | 211 |  |  |
| Observations | 417 |  |  |
| Marginal R^2^ / Conditional R^2^ | 0.374 / 0.693 |  |  |

# Section 8. Main Study, Exploratory Analysis: Perceived Likelihood By Perceived Severity

As in the Pilot Study, we tested the relationship between participants’ subjective severity classifications and their likelihood classifications. We built the following model: lmer(perceived_likelihood ~ perceived_severity*Country + (1|participant). The model's total explanatory power was moderate (conditional R^2^ = 0.19) and the part related to the fixed effects alone (marginal R^2^) was 0.07.

Our analysis showed a significant main effect of Perceived severity, *F*(3,5911.8) =65.9, *p* < .001, *η ^2^* = 3%, as well as a main effect of Country, *F*(3,255.4) = 3.83, *p* = .01, *η ^2^* = 4%, and a significant interaction effect, *F*(9,5902.8) = 4.31, *p* < .001, *ηp2* = 0.6%. For full model output see Table I.

*p p*

**Table I.**

*Full model output for: lmer(perceived_likelihood ~ perceived_severity*Country + (1|Participant).*

| **Perceived likelihood** | | | |
| --- | --- | --- | --- |
| *Predictors* | *Estimates* | *CI* | *p* |
| (Intercept) | 2.29 | 2.17 – 2.42 | **< .001** |
| Perceived severity [minor] | 0.42 | 0.30 – 0.55 | **< .001** |
| Perceived severity [significant] | 0.55 | 0.43 – 0.67 | **< .001** |
| Perceived severity [severe] | 0.58 | 0.44 – 0.71 | **< .001** |
| Country [Malaysia] | 0.18 | -0.18 – 0.54 | .322 |
| Country [Philippines] | -0.20 | -0.44 – 0.04 | .108 |
| Country [Vietnam] | -0.06 | -0.25 – 0.13 | .520 |
| Perceived severity [minor] × Country [Malaysia] | -0.14 | -0.52 – 0.24 | .479 |
| Perceived severity [significant] × Country [Malaysia] | -0.15 | -0.50 – 0.20 | .406 |
| Perceived severity [severe] × Country [Malaysia] | 0.09 | -0.27 – 0.45 | .624 |
| Perceived severity [minor] × Country [Philippines] | 0.02 | -0.22 – 0.26 | .886 |
| Perceived severity [significant] × Country [Philippines] | 0.04 | -0.20 – 0.27 | .751 |
| Perceived severity [severe] × Country [Philippines] | 0.22 | -0.05 – 0.48 | .105 |
| Perceived severity [minor] × Country [Vietnam] | -0.01 | -0.20 – 0.18 | .897 |
| Perceived severity [significant] × Country [Vietnam] | 0.25 | 0.06 – 0.43 | **.008** |

| Perceived severity [severe] × Country [Vietnam] | 0.43 | 0.22 – 0.64 | **< .001** |
| --- | --- | --- | --- |
| **Random Effects** |  |  |  |
| σ2 | 0.72 |  |  |
| τ00 participant | 0.10 |  |  |
| ICC | 0.12 |  |  |
| N participant | 213 |  |  |
| Observations | 5945 |  |  |
| Marginal R^2^ / Conditional R^2^ | 0.075 / 0.185 |  |  |

We tested the relationship between participants’ subjective severity classifications and their likelihood classifications within each country, by building a LMM lmer(perceived_likelihood

~ perceived_severity + (1|participant) for Vietnam, Indonesia, Malaysia, and the Philippines. A main effect of perceived severity was found in the Philippines, *F*(3, 876) = 16.77, *p* < .001, *η ^2^ =* 5%, as well as in Vietnam, *F*(3, 2024.7) = 71.45 *p* < .001, *η ^2^ =* 10%, in Malaysia, *F*(3,

*p p*

637.3) = 7.75 *p* < .001, *η ^2^ =* 4%, and in Indonesia, *F*(3, 2333.4) = 29.11 *p* < .001, *η ^2^ =* 4%.

*p p*

# Section 9: Main Study, Output RQ2 Analysis

The best converging model was: ‘Total Likelihood Score’ ∼ ‘Severity’ ∗ ‘Country’∗ ‘Scenario Type’ + (Scenario Type| participant) + (Severity | participant). Maximal model: ‘Total Likelihood Score’ ∼ ‘Severity’ ∗ ‘Country’∗ ‘Scenario Type’ + (Severity*Scenario Type| participant). Our best converging model’s explanatory power related to the fixed effects alone (marginal *R*^2^) was 0.10. For full analysis output see Table J. See Figure B for graphical representation of mean likelihood classifications by scenario type and country.

**Table J.**

*Output of model: Total Likelihood Score’ ∼ ‘Severity’ ∗ ‘Country’∗ ‘Scenario Type’ + (Scenario Type| participant) + (Severity | participant).*

|  |  | **Total Score** |  |
| --- | --- | --- | --- |
| *Predictors* | *Estimates* | *CI* | *p* |
| (Intercept) | 6.61 | 6.38 – 6.83 | **< .001** |
| Severity [Minor] | -0.36 | -0.61 – -0.11 | **.005** |
| Country [Malaysia] | 0.13 | -0.35 – 0.62 | .593 |
| Country [Philippines] | 0.08 | -0.35 – 0.51 | .713 |
| Country [Vietnam] | 0.11 | -0.22 – 0.44 | .515 |
| Scenario Type [Single Warning] | 0.10 | -0.14 – 0.33 | .434 |
| Severity [Minor] — Country [Malaysia] | 0.49 | -0.05 – 1.03 | .077 |
| Severity [Minor] — Country [Philippines] | 0.23 | -0.24 – 0.71 | .339 |
| Severity [Minor] — Country [Vietnam] | -0.53 | -0.90 – -0.17 | **.004** |
| Severity [Minor] — Scenario Type [Single Warning] | 0.07 | -0.27 – 0.41 | .678 |
| Country [Malaysia] —Scenario Type [Single Warning] | 0.04 | -0.48 – 0.55 | .893 |
| Country [Philippines] — Scenario Type [Single Warning] | -0.13 | -0.58 – 0.33 | .585 |
| Country [Vietnam] — Scenario Type [Single Warning] | 0.13 | -0.21 – 0.48 | .449 |
| (Severity [Minor] — Country [Malaysia]) — Scenario Type [Single Warning] | -0.59 | -1.32 – 0.13 | .110 |
| (Severity [Minor] — Country [Philippines])  — Scenario Type [Single Warning] | -0.13 | -0.78 – 0.51 | .683 |
| (Severity [Minor] — Country [Vietnam]) — Scenario Type [Single Warning] | -0.28 | -0.78 – 0.21 | .261 |

| **Random Effects** |  |
| --- | --- |
| σ2 | 0.62 |
| τ00 participant | 0.26 |
| τ00 participant.1 | 0.23 |
| τ11 participant.SeverityLow | 0.12 |
| τ11 participant.1.Scenario_TypeSingle Warning | 0.00 |
| ρ01 participant | 0.94 |
| ρ01 participant.1 | 1.00 |
| ICC | 0.44 |
| N participant | 213 |
| Observations | 850 |
| Marginal R^2^ / Conditional R^2^ | 0.099 / 0.494 |

**Figure E.**

*Mean likelihood classification in minor and severe impact trials in sequential and single warning scenarios by country.*


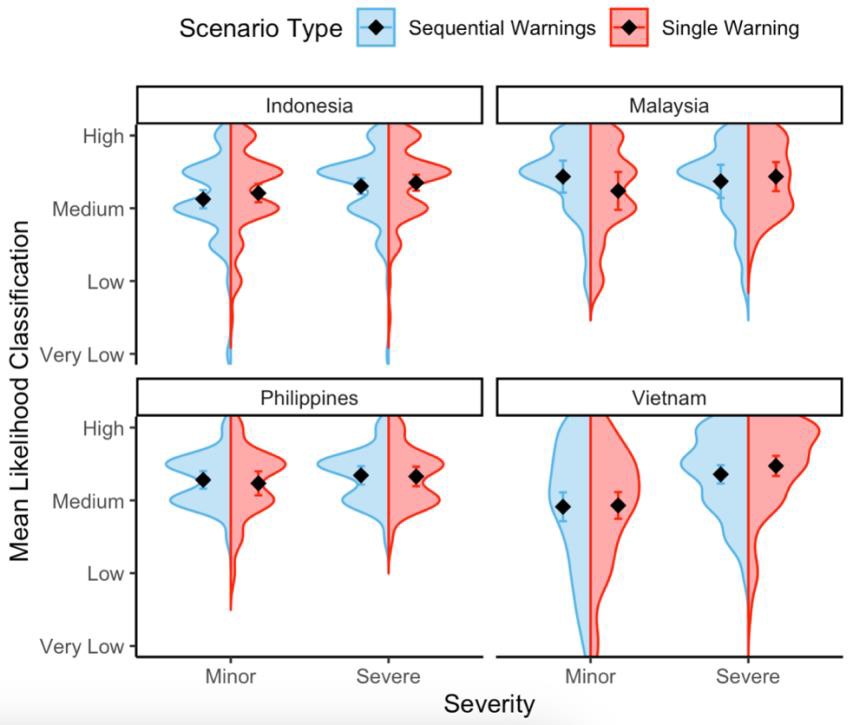


Through post-hoc pairwise comparisons (Tukey HSD corrected) we found a significant difference in the averaged Likelihood ratings relating to low severity scenarios between Malaysia and Vietnam only, *p* = .01. We additionally found a significant difference in the averaged Likelihood ratings relating to high severity scenarios between Indonesia and the Philippines, *p* = .018, Indonesia and Vietnam, *p* < .001, Malaysia and the Philippines, *p* = .009 and the Philippines and Vietnam *p* < .001.

Due to a slight violation of the assumption of normality, as a robustness check, we carried out a paired-samples Wilcoxon Signed-Ranks Test to verify our main findings addressing RQ2. A paired Wilcoxon Signed-Ranks Test confirmed no statistical differences in the scores of participants between single and sequential scenarios, *Z* = 11990.5, *p* = .22.

# Section 10: Main Study, RQ3 Analysis

The best converging model was: ‘Total Likelihood Score’ ∼ ‘T1-Severity ∗ ‘Country’+ (1 | participant). Our best converging model’s total explanatory power (conditional *R*^2^) was

0.84 and explanatory power related to the fixed effects alone (marginal *R*^2^) was 0.04. For full model output, see Table K. Due to a violation of the assumption of normality, Wilcoxon signed- ranks tests confirmed the robustness of our central findings, *Z* = 5440, *p* = .058.

**Table K.**

*Output of Analysis for Model: ‘Total Likelihood Score’ ∼ ‘T1-Severity ∗ ‘Country’+ (1| Participant).*

|  |  | **Total Score** |  |
| --- | --- | --- | --- |
| *Predictors* | *Estimates* | *CI* | *p* |
| (Intercept) | 13.16 | 12.71 – 13.61 | **< .001** |
| Anchor Severity [Minor] | -0.38 | -0.64 – -0.11 | **.006** |

| Country [Malaysia] | 1.06 | 0.08 – 2.03 | **.034** |
| --- | --- | --- | --- |
| Country [Philippines] | -0.35 | -1.21 – 0.51 | .427 |
| Country [Vietnam] | 0.16 | -0.50 – 0.82 | .636 |
| Anchor Severity [Minor]—Country [Malaysia] | 0.20 | -0.37 – 0.77 | .483 |
| Anchor Severity [Minor] —Country [Philippines] | 0.78 | 0.28 – 1.29 | **.002** |
| Anchor Severity [Minor] —Country [Vietnam] | -0.18 | -0.57 – 0.21 | .365 |
| **Random Effects** |  | |  |
| σ2 | 0.75 | |  |
| τ00 participant | 3.69 | |  |
| ICC | 0.83 | |  |
| N participant | 213 | |  |
| Observations | 422 | |  |
| Marginal R^2^ / Conditional R^2^ | 0.037 / 0.837 | |  |

Post-hoc pairwise comparisons (Tukey HSD corrected) demonstrated no significant between- country differences in the summed average Likelihood classifications relating to *severe* impact T1-severity scenarios (all *p*s > 0.05) or relating to minor impact T1-severity scenarios (all *p*s >

.05).

## 10.1: Exploratory Analyses H3

The best converging model was: ‘Total Likelihood Score’ ∼ ‘T1-Severity ∗ ‘Country’+ (1 | participant). Our best converging model’s total explanatory power (conditional *R*^2^) was 0.45 and explanatory power related to the fixed effects alone (marginal *R*^2^) was 0.04. For full model output, see Table L.

**Table L.**

*Output of Analysis for Model: ‘Likelihood Classification T2’ ∼ ‘Perceived T1-Severity ∗*

*‘Country’+ (1 | participant).*

| **T2 Likelihood Rating** | | | |
| --- | --- | --- | --- |
| *Predictors* | *Estimates* | *CI* | *p* |
| (Intercept) | 3.16 | 3.02 – 3.29 | **<0.001** |
| T1 Severity Rating [minor] | 0.07 | -0.05 – 0.19 | 0.256 |
| T1 Severity Rating [significant] | 0.15 | 0.02 – 0.28 | **0.019** |
| T1 Severity Rating [severe] | 0.26 | 0.12 – 0.41 | **<0.001** |
| Country [Malaysia] | 0.29 | -0.13 – 0.71 | 0.178 |
| Country [Philippines] | 0.11 | -0.16 – 0.38 | 0.415 |
| Country [Vietnam] | -0.12 | -0.33 – 0.10 | 0.280 |
| T1 Severity Rating [minor] × Country [Malaysia] | -0.19 | -0.63 – 0.24 | 0.389 |
| T1 Severity Rating [significant] × Country [Malaysia] | -0.09 | -0.51 – 0.32 | 0.659 |
| T1 Severity Rating [severe] × Country [Malaysia] | -0.03 | -0.45 – 0.40 | 0.906 |
| T1 Severity Rating [minor] × Country [Philippines] | -0.04 | -0.29 – 0.20 | 0.736 |
| T1 Severity Rating [significant] × Country [Philippines] | -0.15 | -0.40 – 0.10 | 0.233 |
| T1 Severity Rating [severe] × Country [Philippines] | -0.46 | -0.77 – -0.16 | **0.003** |
| T1 Severity Rating [minor] × Country [Vietnam] | 0.11 | -0.09 – 0.30 | 0.294 |
| T1 Severity Rating [significant] × Country [Vietnam] | 0.16 | -0.04 – 0.37 | 0.116 |
| T1 Severity Rating [severe] × Country [Vietnam] | 0.26 | 0.01 – 0.51 | **0.041** |
| **Random Effects** |  | |  |
| σ2 | 0.26 | |  |
| τ00 participant | 0.19 | |  |
| ICC | 0.43 | |  |
| N participant | 207 | |  |
| Observations | 1651 | |  |
| Marginal R^2^ / Conditional R^2^ | 0.041 / 0.450 | |  |

Post-hoc comparisons revealed that within Indonesia, significant differences in likelihood ratings at T2 were found when T1 perceived severity was minimal vs. severe (*p* = .002) and minor vs. severe (*p* = .02). Within Vietnam, differences were found when T1-perceived severity was minimal vs. significant ( *p* < .001), minimal vs. severe (*p* < .001), minor vs. severe (*p* <

.001) and significant vs. severe (*p* = .04).

Within Malaysia and the Philippines no differences in T2 likelihoods were found depending on the level of perceived T1-severity.

# Section 11: Main Study, Output RQ4 Analysis

The model was: Best Estimate ∼ Severity * Likelihood * Country + (Severity | Participant) + (Likelihood | Participant). The model’s explanatory power related to the fixed effects alone (marginal *R*^2^) was 0.75. The full output of the analysis can be seen in Table M.

**Table M.**

*Output of Model: Best Estimate ∼ Severity * Likelihood * Country + (Severity | Participant)*

*+ (Likelihood | Participant).*

|  |  | **Best Estimate** |  |
| --- | --- | --- | --- |
| *Predictors* | *Estimates* | *CI* | *p* |
| (Intercept) | 27.12 | 22.05 – 32.18 | **<.001** |
| Severity [Severe] | 14.48 | 9.27 – 19.68 | **<.001** |
| Likelihood [Low] | 10.34 | 6.22 – 14.46 | **<.001** |
| Likelihood [Medium] | 33.14 | 28.17 – 38.12 | **<.001** |

| Likelihood [High] | 43.04 | 36.70 – 49.38 | **<.001** |
| --- | --- | --- | --- |
| Country [Malaysia] | -3.51 | -14.43 – 7.41 | .529 |
| Country [Philippines] | -10.24 | -19.89 – -0.60 | **.037** |
| Country [Vietnam] | -7.79 | -15.19 – -0.40 | **.039** |
| Severity [Severe] *Likelihood [Low] | 5.57 | 0.11 – 11.04 | **.046** |
| Severity [Severe] * Likelihood [Medium] | 1.43 | -4.04 – 6.89 | .608 |
| Severity [Severe] * Likelihood [High] | 6.19 | 0.73 – 11.66 | **.026** |
| Severity [Severe] * Country [Malaysia] | -2.91 | -14.14 – 8.32 | .611 |
| Severity [Severe] * Country [Philippines] | -8.29 | -18.20 – 1.62 | .101 |
| Severity [Severe] * Country [Vietnam] | -1.62 | -9.23 – 5.99 | .676 |
| Likelihood [Low] * Country [Malaysia] | 1.27 | -7.61 – 10.15 | .779 |
| Likelihood [Medium] * Country [Malaysia] | -4.06 | -14.78 – 6.67 | .458 |
| Likelihood [High] * Country [Malaysia] | 3.79 | -9.88 – 17.46 | .587 |
| Likelihood [Low] * Country [Philippines] | 8.88 | 1.04 – 16.72 | **.026** |
| Likelihood [Medium] * Country [Philippines] | 14.67 | 5.20 – 24.14 | **.002** |
| Likelihood [High] * Country [Philippines] | 25.40 | 13.33 – 37.47 | **< .001** |
| Likelihood [Low] * Country [Vietnam] | 4.27 | -1.75 – 10.29 | .164 |
| Likelihood [Medium] * Country [Vietnam] | -2.02 | -9.29 – 5.24 | .585 |
| Likelihood [High] *Country [Vietnam] | -3.64 | -12.91 – 5.62 | .441 |
| (Severity [Severe] *Likelihood [Low]) * Country [Malaysia] | -1.57 | -13.36 – 10.22 | .794 |
| (Severity [Severe] *Likelihood [Medium])  *Country [Malaysia] | 9.31 | -2.48 – 21.10 | .121 |
| (Severity [Severe] *Likelihood [High]) * Country [Malaysia] | 1.33 | -10.46 – 13.12 | .825 |

| (Severity [Severe] *Likelihood [Low]) * Country [Philippines] | -3.32 | -13.73 – 7.08 | .531 |
| --- | --- | --- | --- |
| (Severity [Severe] *Likelihood [Medium])  *Country [Philippines] | 4.85 | -5.55 – 15.26 | .360 |
| (Severity [Severe] *Likelihood [High]) * Country [Philippines] | -3.66 | -14.06 – 6.75 | .490 |
| (Severity [Severe] *Likelihood [Low]) * Country [Vietnam] | -6.14 | -14.13 – 1.84 | .132 |
| (Severity [Severe] *Likelihood [Medium])  *Country [Vietnam] | 7.70 | -0.28 – 15.69 | .059 |
| (Severity [Severe] *Likelihood [High]) * Country [Vietnam] | 13.68 | 5.70 – 21.67 | **.001** |
| **Random Effects** |  |  |  |
| σ2 | 163.01 |  |  |
| τ00 participant | 107.01 |  |  |
| τ00 participant.1 | 289.92 |  |  |
| τ11 participant.SeveritySevere | 265.77 |  |  |
| τ11 participant.1.LikelihoodLow | 44.19 |  |  |
| τ11 participant.1.LikelihoodMedium | 213.90 |  |  |
| τ11 participant.1.LikelihoodHigh | 551.60 |  |  |
| ρ01 participant | -1.00 |  |  |
| ρ01 participant.1.LikelihoodLow | -0.70 |  |  |
| ρ01 participant.1.LikelihoodMedium | -0.91 |  |  |
| ρ01 participant.1.LikelihoodHigh | -0.92 |  |  |
| N participant | 213 |  |  |
| Observations | 1704 |  |  |
| Marginal R^2^ / Conditional R^2^ | 0.752 / NA |  |  |

# Section 12: Main Study, RQ5 Exploratory Analyses

**Table N.**

*Contingency table of number of participants whose warnings were in line with a severity effect vs no severity effect, and their risk matrix choice (‘A’ representing a non-severity effect strategy and ‘B’ representing a severity effect strategy).*

|  |  | Risk Matrix A | Risk Matrix B |
| --- | --- | --- | --- |
| Aggregated | Severity Effect | 102 | 30 |
|  | No Severity Effect | 56 | 18 |
| Indonesia | Severity Effect | 40 | 9 |
|  | No Severity Effect | 26 | 5 |
| Malaysia | Severity Effect | 10 | 2 |
|  | No Severity Effect | 9 | 2 |
| Philippines | Severity Effect | 11 | 4 |
|  | No Severity Effect | 15 | 2 |
| Vietnam | Severity Effect | 41 | 15 |
|  | No Severity Effect | 6 | 9 |

**Table O.**

*Contingency table of number of participants whose warnings were in line with a severity effect vs. no severity effect, and their dichotomised Likert Scale rating (severity effect propensity vs. no severity effect propensity).*

|  |  | Severity Effect Propensity | No Severity Effect Propensity |
| --- | --- | --- | --- |
| Aggregated | Severity Effect | 119 | 13 |
|  | No Severity Effect | 60 | 14 |
| Indonesia | Severity Effect | 44 | 5 |
|  | No Severity Effect | 25 | 6 |
| Malaysia | Severity Effect | 10 | 2 |
|  | No Severity Effect | 9 | 2 |
| Philippines | Severity Effect | 13 | 2 |
|  | No Severity Effect | 11 | 6 |
| Vietnam | Severity Effect | 52 | 4 |
|  | No Severity Effect | 15 | 0 |

## Main Study, RQ5 Qualitative Data Analysis

To probe participants’ intuitions regarding the optimality of warnings aligned with a severity effect, we carried out exploratory analyses on qualitative data obtained from the responses of participants to the free-form question in the secondary task asking them to explain when and under what circumstances, adopting a severity effect strategy such as the one exemplified by Risk Matrix B in the scenario, would be useful. Initially, responses obtained from Indonesia and Vietnam were translated to English by RN and SA (Indonesia) and LH, TQD and HN (Vietnam) Subsequently, each participant’s think-aloud response was analysed, taking a data- driven approach to qualitative coding. As such, a rater (AJLH) initially summarised each response using a sentence that best captured its meaning and content, and subsequently categorised these into a set of qualitative codes that were able to represent the key themes of the dataset. Subsequently, SCJ coded all responses using the same coding scheme. 72% of responses were coded identically by the coders. 12% (25) of responses simply reflected SCJ *adding* a coding of ‘reference to impact severity’ to the same codes used by AJLH, where AJLH tended to only use that coding where other codes were not appropriate (given that often the references were direct repetitions of information in the scenario). AJLH and SCJ subsequently agreed to AJLH’s coding for these items. The remaining 15% (33) of responses were resolved through discussion (22/12/2022). For a list of the final themes extracted from the data, the initial codes and example quotes that fit within these, as well as the frequency of the final themes within the aggregated sample, see Table P. Note that participants were told that, if they would never choose Risk Matrix B in this scenario, they could state so in the text box by typing: “I would never choose B”. Finally, note that more than one code and theme could be associated with a participants’ response, thus leaving us with a total of 227 themes associated with participant’s responses.

The analysis showed that the majority of responses (71%) included a code of “never choose Risk Matrix B – a severity effect strategy – for this likelihood”. This is in line with participants’ responses on the dichotomous choice question, but less so with those provided from the Likert scale question which showed that few participants selected ‘Always choose Risk Matrix A’. It should be noted that, included amongst responses classified under the theme of “never choose Risk Matrix B for this likelihood”, were responses stating that this risk matrix might be chosen for a different set of likelihood and severity information than the one stated by the scenario in question. Qualitative coding also revealed that “preparedness/precautionary” – describing reasoning relating to believing a severity effect strategy might be useful to communicate severe situations and trigger more attention and responsiveness to the event –was present in 7% of participant responses. In addition, a theme of “impact severity” was present in 12% of codes. This theme represented reasoning that focused on the high severity of the impact, and the high exposure and vulnerability that can arise from this, when justifying why a severity-effect strategy might be useful.

**Table P.**

*Themes, initial codes, excerpts from responses and frequency of themes in aggregated sample.*

| Final Themes | Initial codes | Example quote | Frequency |
| --- | --- | --- | --- |
| Would never choose B for this likelihood | - Never choose B - If impact and likelihood as indicated on matrix - Communicating with high-risk areas | *I will never choose B because the likelihood is low.* | 152 |
| Reference to timepoint or changing impact | - Near timepoints - Early timepoints - Suggests impact will worsen | *I would choose warning choice B if the event*  *is 24 hours*  *ahead already. If choice A is 3 days from the event and Choice B is 24 hours prior to the event.* | 10 |

| Reference to severe impact events usually paired with higher probs | - This happens quite often - Warning often pushed one level higher than expected - Severe impact only occurs for higher probs | *Because warning will often be pushed 1 level higher*  *than expected* | 8 |
| --- | --- | --- | --- |
| Preparedness/ Precautionary | - To enable preparedness/attention - Communicate worst case scenario | *Even if likelihood is low, appropriate*  *preparation is important if you know*  *that impact is severe.* | 15 |
| Reference to impact severity | - If it suggests impact will worsen - High exposure / vulnerability - High / severe impact | *I'll choose B if the affected*  *are the people or may relate to the lives of the people.* | 25 |
| Lack of trust in model | - Error of model - If medium impact already observed | *I may choose warning choice*  *B if there is already a report on the medium impact upon the formulation of the warning.* | 3 |
| Other | - Win/win - Low risk - Hazard specific - No identifiable response - Other |  | 22 |

# Section 13: Main Study Exploratory Analyses

## Professional Experience And The Severity Effect

In an exploratory analysis, we investigated whether experience with impact-based weather forecasting (IBF), and/or experience utilising risk matrices, influenced participants’ use of a severity effect strategy when making weather warnings in the primary task. Within the aggregated sample, 3.3 % of participants stated they had never heard of IBF, 27.7 % stated they had heard of it, 20.1 % stated they received training on it, and 25.5 % stated they used it before

(in either work or training exercises) and 23.4 % stated they use it regularly. In addition, within the aggregated sample, 11.3 % of participants stated never having seen a risk matrix before,

31.9 % stated they had seen it but never used it, 23.5 % stated they received training on it and

33.3 % stated they used it before in their work or studies (see Table 2 Main Text). Participants’ responses to the experience questions were standardised as *z*-scores and were analysed separately given a lack of sufficient consistency (Cronbach’s alpha = .67).

## Experience With IBF and the Severity Effect

There was no indication that experience with IBF reduced the Severity Effect (Figure F). A two-way (IBF experience × Country) non-parametric Scheirer Ray Hare test demonstrated a main effect of Country, *H* (3, 186) = 34.19, *p* < .001, no main effect of experience with IBF, *H* (4,186) = 8.71, *p* = .068, and no significant interaction effect *H* (12,186) = 8.13, *p* = .77.

**Figure F.**

*Boxplot of severity scores by IBF experience, in each country sample.*


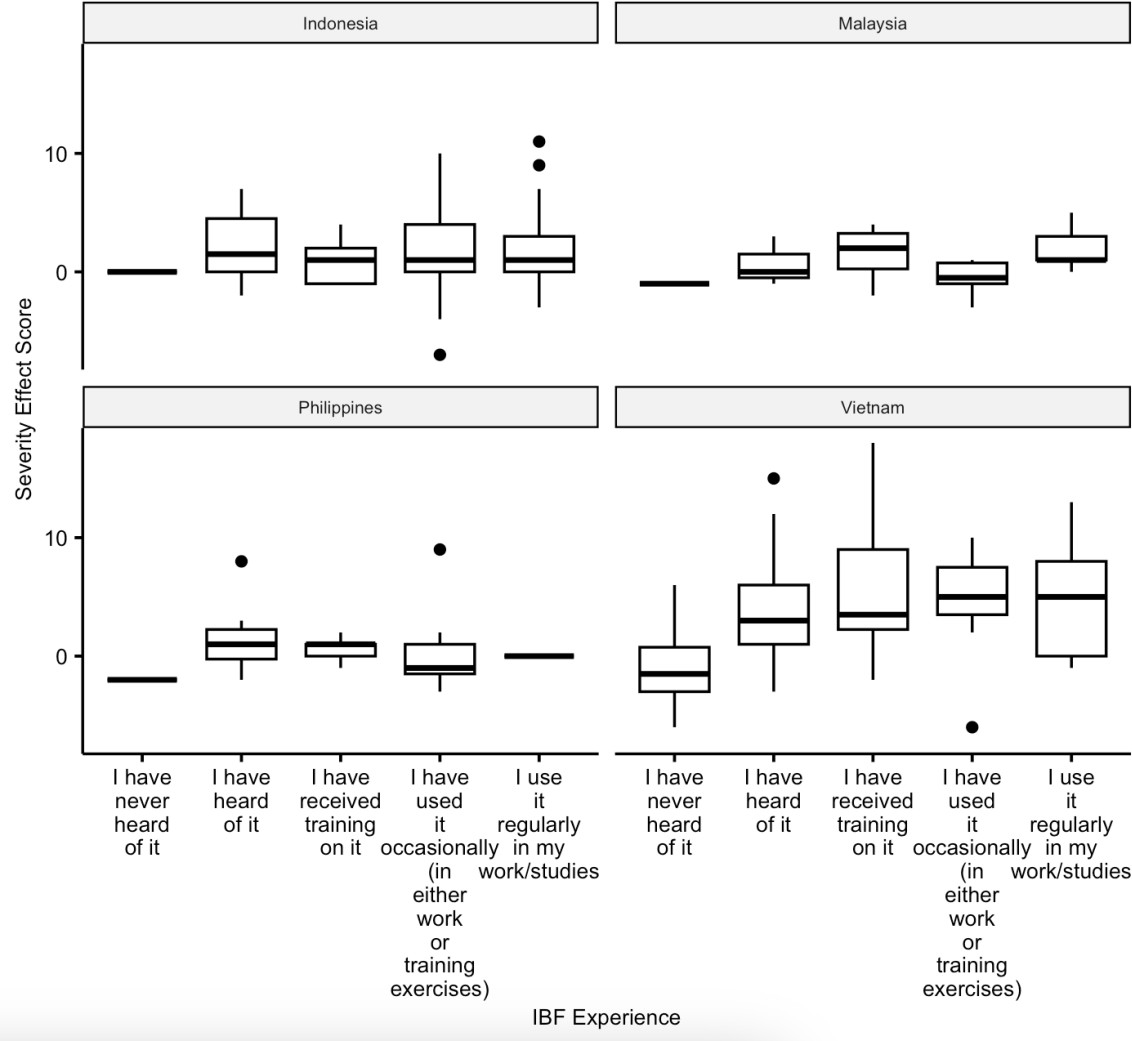


## Experience With Risk Matrices and the Severity Effect

There was no indication that experience with risk matrices was associated with the prevalence of the Severity effect (Figure G). A two-way (Risk Matrix Experience × Country) non- parametric Scheirer Ray Hare test revealed a main effect of Country, *H* (3, 190) = 23.3, *p* <

.001, no main effect of risk matrix experience, *H* (3,190) = 3.82, *p* = .28, and no significant interaction effect *H* (9,190) = 16.37, *p* = .059.

**Figure G.**

*Boxplot of severity scores by Risk Matrix experience, in each country sample.*


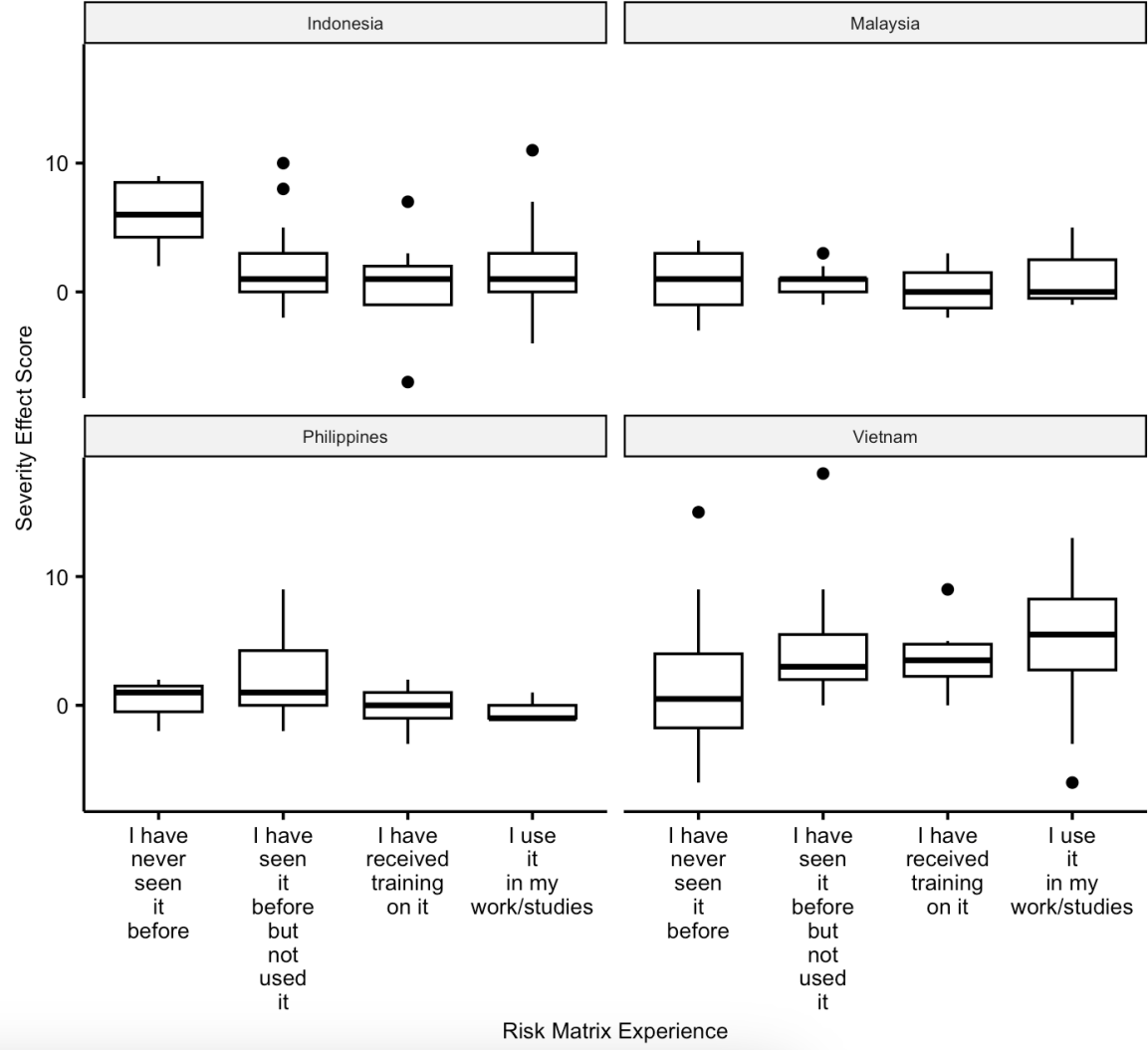


# Section 14: Pilot Study Impact Tables

**Table Q.**

*Selected impacts for a ‘heavy rainfall’ hazard in Indonesia.*

| **Minimal** | **Minor** | **Significant** | **Severe** |
| --- | --- | --- | --- |
| 1. Some pooling of water on roads or  in informal settlements. | Minor motor vehicle accidents due to slippery roads. | Displacement of affected communities. | Widespread flooding of settlements. |
| Day to day activities not disturbed. | Wet roads and reduced visibility. | Possible damage to roads and bridges. | Widespread, prolonged disruption to essential services (water, electricity, etc). |
| Major roads affected but can be used, longer travel times. | Localized and short-term disruption to municipal services (water, electricity, hospitals, schools, etc). | Disruption of access to drinking water. | Widespread transport routes and travel services severely affected. |
| Minimal traffic congestion. | Coastal inundation (due to combination of rain, water level, and wind). | Isolated incidents of communicable diseases. | Widespread damage to property and buildings. |

**Table R.**

*Selected impacts for a ‘heavy rainfall’ hazard in the Philippines.*

| **Minimal** | **Minor** | **Significant** | **Severe** |
| --- | --- | --- | --- |
| 1. Temporary class disruption (few hours to a day or two). | 6. Temporary class suspensions (few hours to a day or two). | 11. Damage to educational infrastructure. | 16. Widespread damage to powerlines and pipelines. |
| 2. Incidences of non- life-threatening illnesses. | 7. Contaminated water supply for hours to a few days, minor | 12. Some cases of communicable disease, emotional and  psychological traumas. | 17. Communicable and waterborne diseases epidemic |

|  | discolouration of water. |  | (leptospirosis etc) and multiple deaths. |
| --- | --- | --- | --- |
| 3. Incidences of exposure to cold and incidents of patients with colds. | 8. Interruption of water, power and communication services for a few days. | 13. Roads not passable to both light and medium vehicles. | 18. Widespread and prolonged contamination of water supply, discolouration of water with pungent odour. |
| 4. Roads passable to all types of road vehicles. | 9. Possible waterborne diseases (e.g. Leptospirosis). | 14. Ongoing limited access to aid to affected. communities for periods of time. | 19. Prolonged food unavailability due to hampered supply of food (more than two weeks). |
| 5. No damage to buildings/structures. | 10. Slight damage (less than 20%) to  buildings/structures. | 15. Partial damage (20-60%) of crops and livestock. | 20. Total damage to buildings/structures. |

# Section 15: Main Study Impact Tables

**Table S.**

*Selected impacts for a ‘heavy rainfall’ hazard in the Manila area, Philippines and Hanoi, Vietnam.*

| **Minor** | **Significant** | **Severe** |
| --- | --- | --- |
| Some traffic congestion. | Pre-school to High school are suspended. | All levels of classes are  suspended, including graduate schools. |
| Flooding may be minimal and localize. | Roads Not passable to light vehicles. | Roads Not passable to all types of vehicles. |
| Continuous operations of  commercial/ manufacturing companies. | Some disruption/power  outage in high-risk areas. | Widespread disruption/power outage for an extended period. |
| Minor impacts on air travel. | Some reports of water- borne disease  incidences. | Widespread water-borne Diseases or illnesses. |

| Minor damages to some infrastructures especially made of light materials. | Some damages to infrastructure like  bridges, roadways. | Water pipelines failure. |
| --- | --- | --- |
| Minimal damages to livestock, crops. | Some damage to livestock, crops. | Overwhelmed healthcare  facilities and personnel due to patient surge in hospitals. |
| Possible or isolated cases of communicable diseases. | Some reports of communicable diseases. | Serious interruption of  delivery of basic services. |
| Possible intermittent/isolated loss of signal connectivity. | Communication will be  downed in high-risk areas. | Road closure and road transport affected. |

**Table T.**

*Selected impacts for a ‘heavy rainfall’ hazard in the Jakarta area, Indonesia.*

| **Minor** | **Significant** | **Severe** |
| --- | --- | --- |
| Coastal inundation (due to combination of rain, water level, and wind). | Localized flooding of susceptible formal/informal settlements or roads, low- lying areas and bridges. | Widespread flooding of roads and settlements. |
| Day to day activities not disturbed. | Isolated mudslides, landslides  and rock falls. | Widespread mudslides and rock falls and soil erosion. |
| Localized and short-term disruption to municipal services (water, electricity, hospitals, schools, etc.) | Disruption of access to drinking water and damage to crops. | Widespread disruption of access to drinking water and damage to crops. |
| Minor motor vehicle accidents due to slippery roads. | Isolated incidents of communicable diseases. | Widespread incidents of communicable diseases. |
| Business as usual | Short term strain on emergency personnel. | Airport closures. |
| Some pooling of water on roads or  in informal settlements. | Flooding of roads and  settlements (formal/informal). | Large communities not  accessible/cut-off for a prolonged period. |

| Minimal traffic congestion. | Major disruption of traffic  flow due to major roads being flooded or closed. | Danger to life (fast flowing streams I deep water. |
| --- | --- | --- |
| Major roads affected but can be used, longer travel  times. | Closure of roads crossing low water bridges. | Major roads and bridges damaged or washed away. |

**Table U.**

*Selected impacts for a ‘river flooding’ hazard in the Kelantan area, Malaysia.*

| **Minor** | **Significant** | **Severe** |
| --- | --- | --- |
| Some minor  Injuries. | Access is limited (road/rail). | Major roads/highways/rail  cut-off. |
| Trees obstructing road/rail. | Large numbers of residential  properties damaged, and some destroyed. | Widespread destruction of  properties and homelessness. |
| Slight delayed flights. | Some roads damaged from floods. | Widespread complete destruction of roads  (major/widespread). |
| Slight shortage of food supply (e.g., rice). | Difficulty obtaining medicine/hospital  availability. | National shortage of medicine. |
| Shortage of clean water and medicine (short period) due to infrastructure disruption. | Issues accessing clean water for prolonged period. | Agricultural land degradation. |
| Temporary disruption to water, electricity, and communication services to  limited areas. | disruption to water, electricity, and communication services to  several areas for several days. | Widespread water borne diseases. |
| Some loss/disruption crop/livestock. | Loss of crops/livestock. | Bridge(s) complete structural destruction  (washed away). |
| Minimal disruption to public services such as schools, healthcare services, district offices in  a few areas. | Major disruption to public services such as schools, healthcare services, district offices in a few areas. | Total destruction of rain gauge and water level instruments. |

# Section 16: Main Study Recruitment Email to Philippines

Email sent from our partner within PAGASA, to their colleagues in the organisation asking them to participate in our survey.

*Dear* ***PAGASA colleagues****,*

*We hope this email finds you will.*

*We wish to inform you that, the University College London (UCL), in partnership with the UK Met Office and PAGASA will be conducting another study focusing on the* ***"Investigation of the Severity Effect in Dynamic Impact-Based Weather Forecasting Scenarios"****, as part of the activities under the* ***WCSSP-SeA Project*** *or Weather and Climate Science for Service Partnership-Southeast Asia particularly under Work Package 3 which develops Impact- Based Forecasting and Warning System. The study will attempt to address research questions as follows:*

1. *Does a ‘severity bias’ manifest itself when making sequential warning choices related to the same event unfolding over time?*
2. *What are the consequences of a ‘severity bias’ when making sequential weather warning choices related to the same event unfolding over time?*
3. *Are participants aware of the severity effect?*
4. *Do participants believe the severity effect is desirable?*
5. *What are the downstream consequences of the severity effect?*

*In view of this and in order to realize the objectives of the study, we would like to invite ALL of you to participate in the survey developed by UCL experts. We hope you can spare a little of your time to answer the survey for our advantage. Results of which will be of great help as we endeavor to transform our early warning services into Impact-Based Forecast and Warnings.*

*SURVEY LINK: Online Survey Software | Qualtrics Survey Solutions Thank you very much.*

1. Corresponding to Very low likelihood of Significant impacts and medium likelihood of Significant impacts. [↑](#footnote-ref-1)
2. Corresponding to Low likelihood of Severe impacts and High likelihood of Severe impacts. [↑](#footnote-ref-2)
